# *DGW1*, encoding an hnRNP‐like RNA binding protein, positively regulates grain size and weight by interacting with *GW6* mRNA

**DOI:** 10.1111/pbi.14202

**Published:** 2023-10-20

**Authors:** Lingfeng Li, Jijin Li, Keke Liu, Chenglong Jiang, Wenhu Jin, Jiangkun Ye, Tierui Qin, Binjiu Luo, Zeyu Chen, Jinzhao Li, Fuxiang Lv, Xiaojun Li, Haipeng Wang, Jinghua Jin, Qiming Deng, Shiquan Wang, Jun Zhu, Ting Zou, Huainian Liu, Shuangcheng Li, Ping Li, Yueyang Liang

**Affiliations:** ^1^ State Key Laboratory of Crop Gene Exploration and Utilization in Southwest China Sichuan Agricultural University Chengdu China; ^2^ Rice Research Institute, Sichuan Agricultural University Chengdu China; ^3^ Neijiang Academy of Agricultural Science in Sichuan Province Neijiang China

**Keywords:** rice, grain size, *DGW1*, RNA‐binding protein, *GW6*

## Abstract

Grain size and weight determine rice yield. Although numerous genes and pathways involved in regulating grain size have been identified, our knowledge of post‐transcriptional control of grain size remains elusive. In this study, we characterize a rice mutant, *decreased grain width and weight 1* (*dgw1*), which produces small grains. We show that *DGW1* encodes a member of the heterogeneous nuclear ribonucleoprotein (hnRNP) family protein and preferentially expresses in developing panicles, positively regulating grain size by promoting cell expansion in spikelet hulls. Overexpression of *DGW1* increases grain weight and grain numbers, leading to a significant rise in rice grain yield. We further demonstrate that DGW1 functions in grain size regulation by directly binding to the mRNA of *Grain Width 6* (*GW6*), a critical grain size regulator in rice. Overexpression of *GW6* restored the grain size phenotype of *DGW1*‐knockout plants. DGW1 interacts with two oligouridylate binding proteins (OsUBP1a and OsUBP1b), which also bind the *GW6* mRNA. In addition, the second RRM domain of DGW1 is indispensable for its mediated protein‐RNA and protein–protein interactions. In summary, our findings identify a new regulatory module of DGW1‐GW6 that regulates rice grain size and weight, providing important insights into the function of hnRNP‐like proteins in the regulation of grain size.

## Introduction

Increasing rice grain yield to feed the growing population is an urgent challenge in the future. Grain size, positively associated with grain weight, is critical in determining rice yield (Xing and Zhang, 2010). Manipulating grain size can improve rice yield and appearance quality (Wang *et al*., 2015a,b; Zhao *et al*., 2018). Thus, grain size has long been a significant target of domestication and breeding in rice.

Increasing genetic evidence suggests that gene regulatory networks derived from multiple cellular signalling pathways are involved in rice grain size regulation, including G protein signalling (Fan *et al*., 2006; Liu *et al*., 2018; Sun *et al*., 2018), mitogen‐activated protein kinase (MAPK) cascades (Duan *et al*., 2014; Guo *et al*., 2018; Liu *et al*., 2015; Tian *et al*., 2021; Xu *et al*., 2018), ubiquitin‐proteasome system (Hao *et al*., 2021; Huang *et al*., 2017; Shi *et al*., 2019; Song *et al*., 2007; Weng *et al*., 2008), phytohormone signalling (Ishimaru *et al*., 2013; Lyu *et al*., 2020; Tong *et al*., 2012; Xiao *et al*., 2019; Zhang *et al*., 2021), and transcription regulation factors (Liu *et al*., 2022a; Si *et al*., 2016; Wang *et al*., 2015a, 2019). It has been reported that some grain size regulators in rice involve multiple signalling pathways. For example, *GL3.1*, encoding a Ser/Thr phosphatase, is a negative regulator of rice grain size and mediates the crosstalk between cytokinin (CK) and brassinolide (BR) signalling (Liu *et al*., 2022b; Qi *et al*., 2012). SMALL ORGAN SIZE 1 (SMOS1) and SMALL ORGAN SIZE 2 (SMOS2) /DWARF AND LOW‐TILLERING (DLT) complexes integrate auxin and BR signals to regulate grain size in rice (Hirano *et al*., 2017). *Grain Width 6* (*GW6*) encodes a GA‐stimulating transcript (GAST) protein and functions as a vital coordinator node among BR, gibberellin (GA) and abscisic acid (ABA) signals, positively regulating rice grain size (Li *et al*., 2021; Shi *et al*., 2020; Wang *et al*., 2009).

RNA binding proteins (RBPs) have been implicated in the post‐transcriptional regulation of gene expression by modulating RNA metabolism processes, such as pre‐mRNA alternative splicing, RNA decay, transport and translation (Xue *et al*., 2021). RBPs interact with target RNAs to form ribonucleoprotein complexes that govern the post‐transcriptional processing of RNA for fine‐tuning the regulation of gene expression in a spatiotemporal manner, consequently controlling diverse physiological processes in plant growth and development (Yeap *et al*., 2014). In higher plants, the oligouridylate binding protein 1 s (UBP1s) have been characterized as hnRNP‐like RBPs that play pivotal roles in intron recognition, pre‐mRNA splicing, mRNA accumulation and translation regulation. NpUPB1 promotes splicing efficiency by enhancing intron recognition of mRNA and protects mRNA from extranuclear degradation by binding to its 3’UTR in *Nicotiana plumbaginifolia* (Lambermon *et al*., 2000). AtUBP1B regulates heat stress tolerance by protecting RNA degradation in *Arabidopsis* (Nguyen *et al*., 2016). AtUBP1C regulates hypoxic stress tolerance in *Arabidopsis* by regulating mRNA translation (Sorenson and Bailey‐Serres, 2014). Two rice RNA binding proteins, RNA Binding Protein P (RBP‐P) and RNA Binding Protein L (RBP‐L), bind to glutelin and prolamine mRNA enabling proper mRNA transport in rice endosperm (Tian *et al*., 2018, 2019a, 2020). *OsLa1* and *OsLa2* are homologues of *Arabidopsis* La protein, which positively regulate grain size and pollen fertility (Guo *et al*., 2022). *LARGE GRAIN* (*LGG*), encoding an hnRNP‐like RBP with two RRMs, regulates grain length in rice (Chiou *et al*., 2019). In addition, *LARGE1*, a putative RBP, which encodes MEI2‐LIKE PROTEIN 4 (OML4) containing three RNA recognition motifs (RRMs), modulates grain size and weight by constraining cell expansion in spikelet hulls (Lyu *et al*., 2020). However, the mechanism by which RBPs bind to their target RNAs to regulate rice grain size remains unclear.

In this study, we report a rice mutant, *decreased grain width and weight 1* (*dgw1*), with decreased grain width and weight. MutMap method reveals that *DGW1* encodes an hnRNP‐like RBP with two RRM domains. We show that *DGW1* was highly expressed in rice panicles and positively regulates grain size by promoting cell expansion in spikelet hulls. Overexpression of *DGW1* in rice increased grain number and size, resulting in a significant grain yield improvement. Furthermore, we demonstrate that DGW1 can physically interact with two oligouridylate binding proteins, OsUBP1a and OsUBP1b, to form potential RBP complexes, which directly bind to the mRNA of a rice grain‐size regulator GW6. Loss of *DGW1* function in rice resembles the phenotypes of *GW6* deficient mutant in grain size and responses to BR and GA. Increased expression of *GW6* in *DGW1*‐knockout plants effectively restored the grain size phenotype. Our work sheds light on the mechanisms of hnRNP‐like protein DGW1 regulating rice grain size and weight by targeting *GW6* mRNA.

## Results

### Rice mutant *dgw1* shows pleiotropic defects in grain size and plant architecture

**Figure 1:**
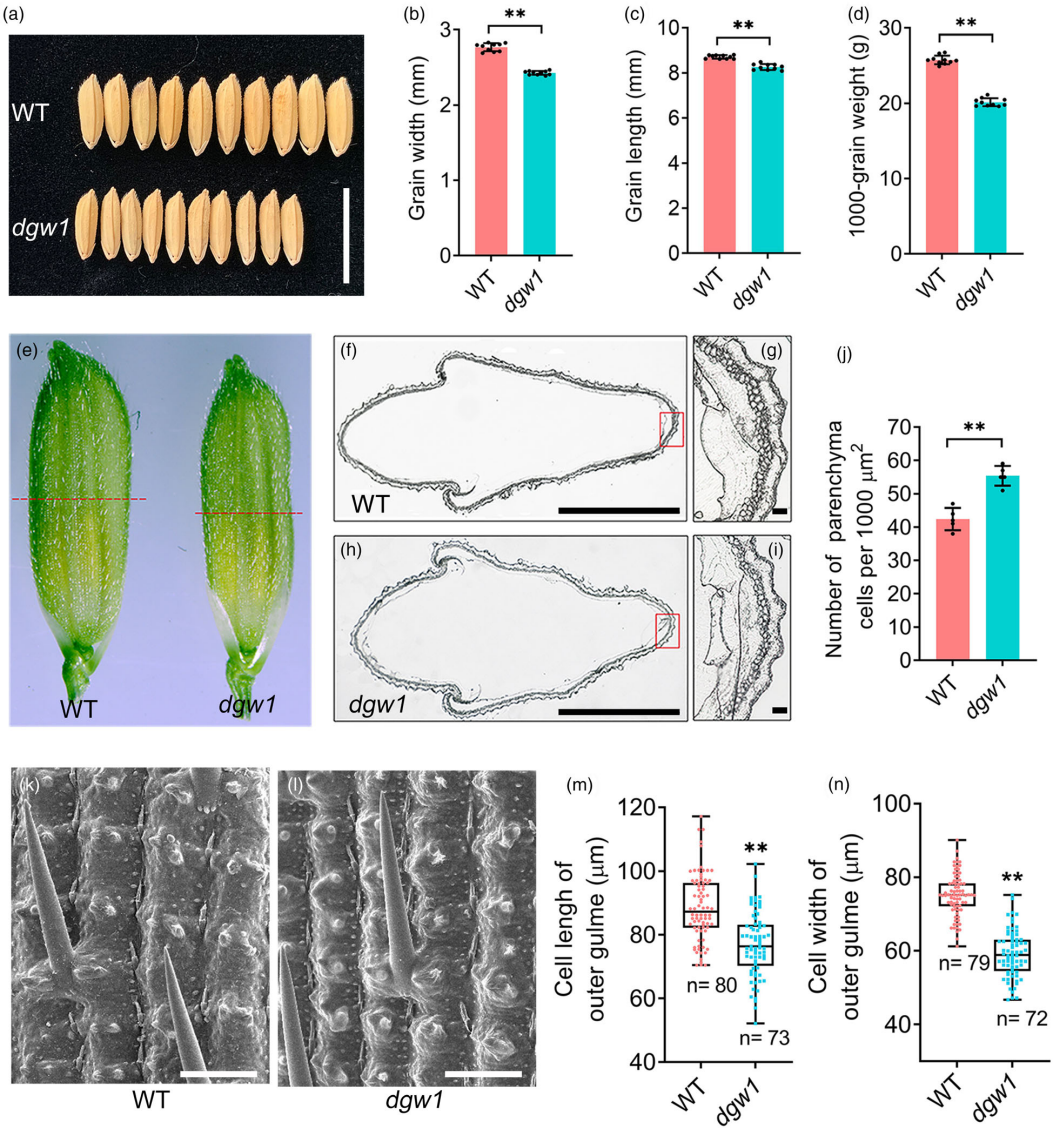
*DGW1* regulates grain size by altering cell expansion. (a) Mature grains of wild‐type (WT) and *dgw1*. Bar = 1 cm. (b, d) Grain width (b), grain length (c) and 1000‐grain weight (d) of WT and *dgw1*. Values are means ± SD (*n* = 10). (e) Spikelets of WT and *dgw1* before athesis. The red line indicates the cross‐section position. (f, h) Cross‐sections of WT (f) and *dgw1* (h) spikelet hulls. Bars = 1 mm. (g, i) Magnified views of the cross‐sections boxed in (f) and (h), respectively. Bars = 10 μm. (j) Number of parenchyma cells per 1000 μm^2^ in WT and *dgw1*. Values are means ± SD (*n* = 5). (k, l) SEM analysis of the outer surfaces of WT (k) and *dgw1* (l) glumes. Bars = 100 μm. (m, n) Outer epidermal cell length (m) and cell width (n) of WT and *dgw1* glumes. Values are means ± SD (*n* > 70). **P < 0.01 compared with the WT using Student's *t*‐test.

Through a large‐scale screen of rice ethyl methane sulfonate–mutagenized M_2_ library in the background of an *indica* rice cultivar Shuhui600 (R600), we isolated a mutant *dgw1*, which exhibited significantly reduced grain width, length and 1000‐grain weight by 12.34%, 5.1% and 21.82%, respectively, compared to the wild‐type (Figure 1a–d). In addition, the dehulled grains of *dgw1* were smaller than that of wild‐type (Figure S1a–d). Regarding panicle morphology, *dgw1* produced the shorter panicle length with fewer primary branches and grains per panicle (Figure S1e–h). The *dgw1* plants displayed a reduction in plant height and an increase tillering, as well as thinner stems and smaller leaf angles compared to the wild‐type (Figure S1i–p). These results indicated that *DGW1* plays a pleiotropic role in regulating both grain size and plant architecture.

### *DGW1* regulates cell expansion in spikelet hulls

Grain size is determined by cell proliferation and cell expansion in rice spikelet hulls (Li *et al*., 2018). The spikelet hulls of *dgw1* were significantly slender than those of the wild‐type plants (Figure 1e). To investigate the cellular mechanism underlying *DGW1*‐mediated regulation of grain size, we analysed the histological cross‐sections of pre‐anthesis spikelet hulls. Compared with the wild‐type plants, the *dgw1* mutant showed the smaller size of inner parenchyma cells and increased cell number per 1000 μm^2^ (Figure 1f–j). The scanning electron microscopy (SEM) observation revealed that the outer epidermal cells in *dgw1* spikelet hulls were narrower and shorter than those in the wild‐type, indicating that the reduction of *dgw1* grain size was primarily due to the limited cell expansion (Figure 1k–n). We also observed a significant reduction in parenchyma cell size of *dgw1* in the third internodes compared to that of the wild‐type (Figure S2). These results indicated that DGW1 modulates grain size and plant height by regulating cell expansion.

### *DGW1* encodes an hnRNP‐like RNA binding protein with two RRM domains

**Figure 2:**
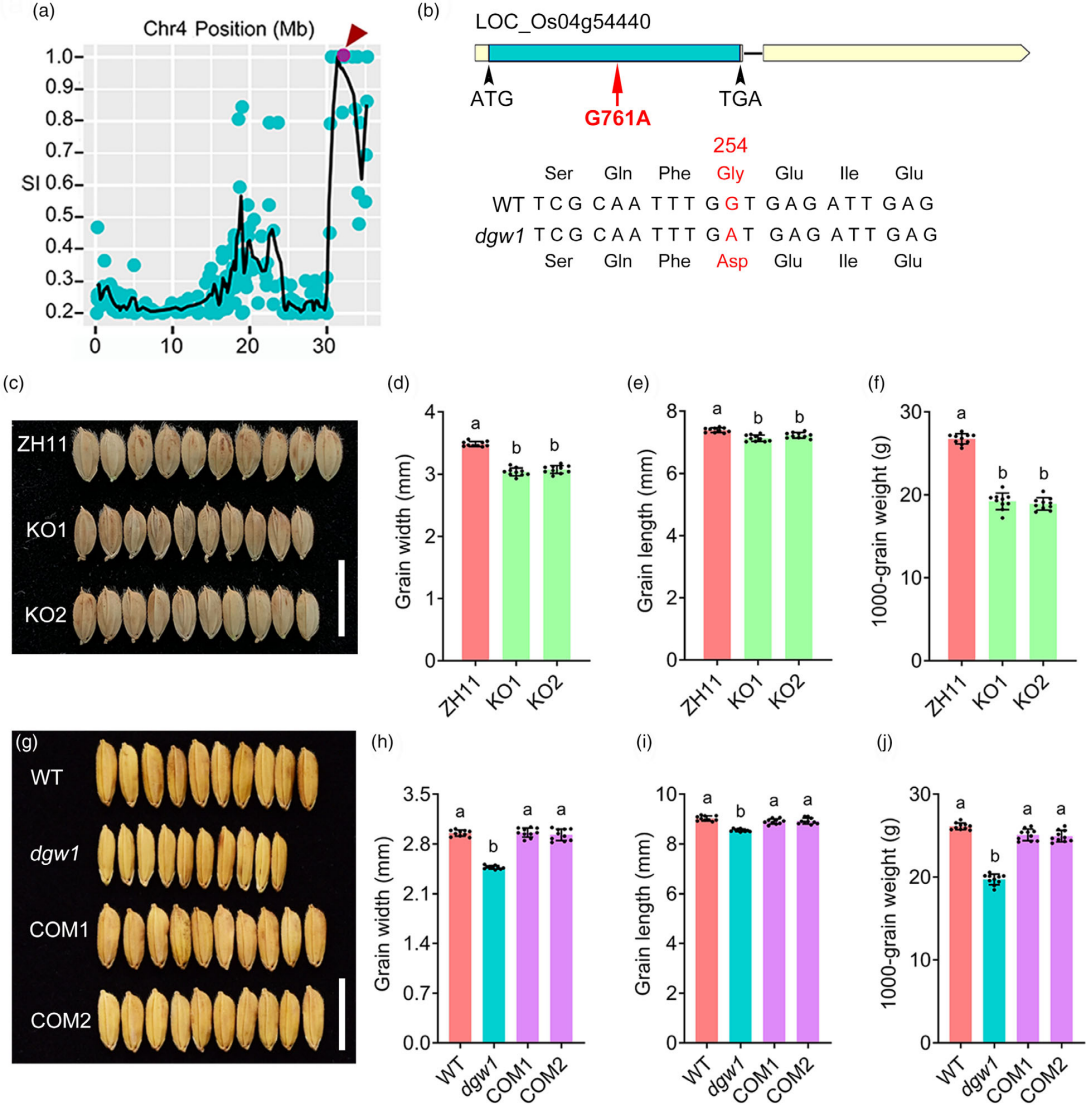
Identification of *DGW1*. (a) Distributions of the SNP index on chromosome 4. The red arrow indicates the candidate mutation site. SI, SNP index. (b) A schematic representation of the *LOC_Os04g54440* gene, and genomic sequences around the mutated position of WT and *dgw1*. The red arrow indicates the *dgw1* mutation site. Blue box, coding sequence; yellow boxes, untranslated regions; intervening lines, introns. The *dgw1* mutation is highlighted in red colour. (c) Mature paddy grains of ZH11, KO1 and KO2. Bar = 1 cm. (d‐f) Comparison of grain width (d), grain length (e) and 1000‐grain weight (f) between ZH11 and KO lines. Values are means ± SD (*n* = 10). Different lowercase letters indicate significant differences between ZH11 and other lines (Student's *t*‐test, *P* < 0.05). (g) Mature paddy grains of WT, *dgw1* and two complemented transgenic lines. Bar = 1 cm. (h‐j) Comparison of grain width (h), grain length (i) and 1000‐grain weight (j) of WT, *dgw1*, COM1 and COM2. Values are means ± SD (*n* = 10). Different lowercase letters indicate significant differences between WT and other lines (Student's *t*‐test, *P* < 0.05).

In order to isolate *DGW1*, we crossed the *dgw1* mutant with its wild‐type counterpart R600 to generate an F_2_ population. The phenotypes of F_1_ heterozygous plants were similar to that of the wild‐type. Analysis of the F_2_ population showed that the ratio of plants exhibiting wild‐type and mutant phenotypes was close to 3: 1 (293: 107; *χ* ^2^ = 0.56, *P* > 0.05), indicating that the phenotypes of *dgw1* were controlled by a single recessive gene. We utilized the MutMap approach to identify the *DGW1* gene (Abe *et al*., 2012). Through analysing bulk sequencing data of small grain size individuals from the F_2_ population, we identified a candidate region at the end of the long arm of chromosome 4 harbouring three single nucleotide polymorphisms (SNPs) in the genes coding regions (Figure 2a). Only one SNP located at *LOC_Os04g54440* was found to be co‐segregated with the *dgw1* grain phenotype, resulting in an amino acid substitution from Gly_254_ (GGT) to ASP (GAT) (Figure 2b). Therefore, we designated *LOC_Os04g54440* as the putative candidate gene for *DGW1*.

To confirm that *LOC_Os04g54440* is actually *DGW1*, we used the CRISPR/Cas9 system to knock out the gene in the ZH11 background. We obtained multiple transgenic plants with diverse mutations at the target site and selected two lines harbouring 1‐bp insertion (KO1) and 8‐bp deletion (KO2), respectively, for further analysis (Figure S3a). Compared with ZH11, both KO lines exhibited decreased grain size and weight (Figure 2c–f) and also resembled the *dgw1* mutant in plant architecture (Figure S3b–h). Histological analysis revealed that the KO lines exhibited reduced cell size in the glume and stem tissues compared to ZH11 (Figure S4). We further conducted a genetic complementation assay by transforming a fragment containing the genomic sequence of *LOC_Os04g54440* and its 2617‐bp promoter region from the wild‐type plants into the *dgw1* mutant. The full‐length genome fragment of wild‐type *LOC_Os04g54440* successfully rescued phenotypic defects of *dgw1* mutant in grain size, weight and plant architecture (Figure 2g–j; Figure S5).

Sequence analysis showed that *DGW1* encodes an hnRNP‐like protein with two RRM domains and belongs to UBP1‐associated (UBA) protein family (Figure S6a,c). Modelling of protein secondary structure indicated that the *dgw1* mutation possibly resulted in a conformational change of the RRM2, suggesting that the mutation may impair protein function (Figure S6b). Phylogenetic analysis revealed that DGW1 homologues are widespread throughout monocots and dicots, and DGW1 forms a small clade with 10 other gramineous homologues (Figure S6c).

### *DGW1* is highly expressed in developing panicles and encodes a nuclear‐localized protein

**Figure 3:**
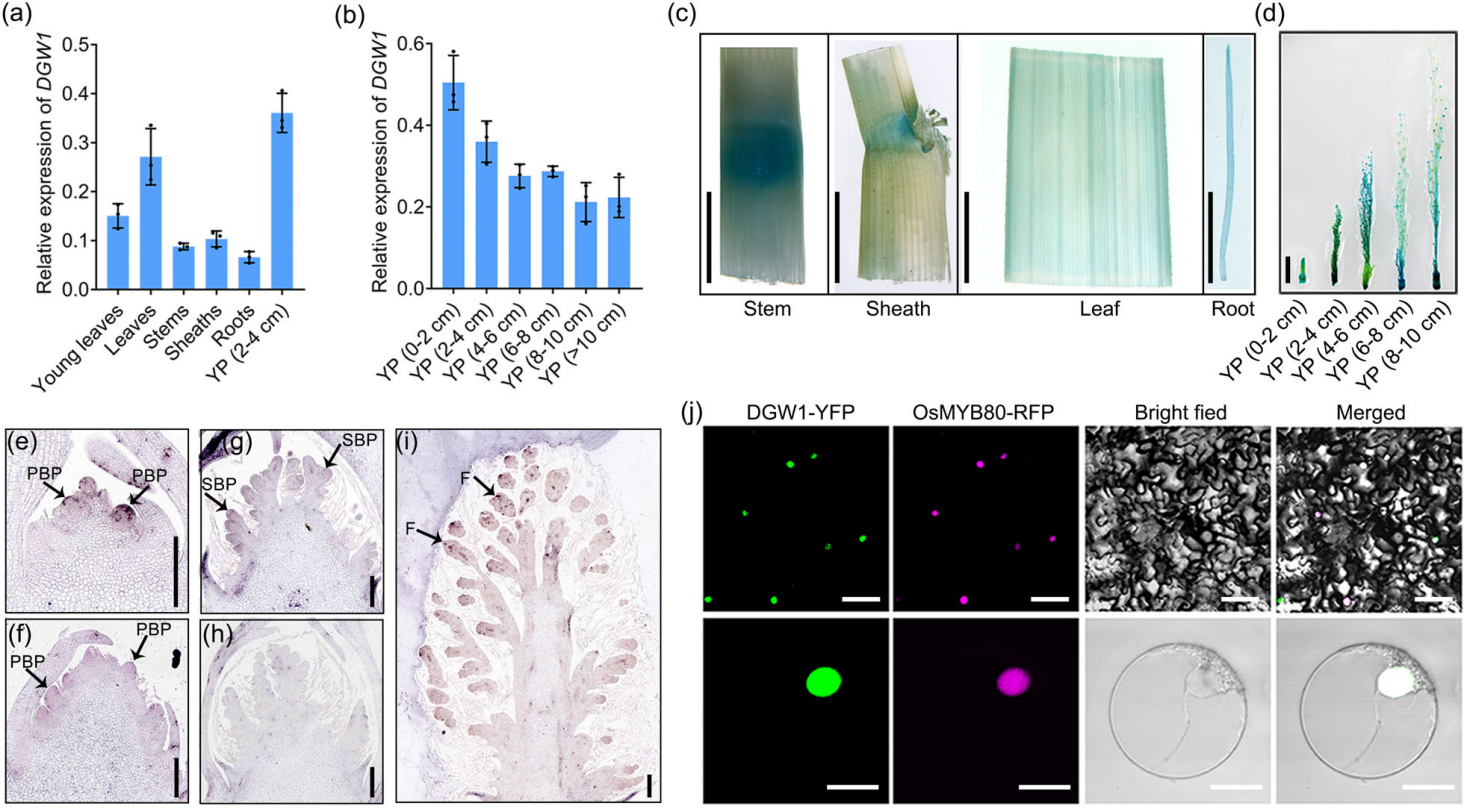
Expression pattern of *DGW1* gene and subcellular localization of DGW1 protein. (a, b) The mRNA levels of *DGW1* in different organs were analysed by RT‐qPCR. YP, young panicles. Rice *ACTIN* was used as a control. Values are means ± SD (*n* = 3). (c, d) Expression of *DGW1* was monitored by *proDGW1::GUS* transgenic plants. GUS activity in stem, sheath, leaf, root and developing panicles. Bars = 1 cm. (e‐i) RNA *in situ* hybridization revealed that *DGW1* is preferentially expressed in primary branch primordia (PBP), secondary branch primordia (SBP) and floret (F). Arrows indicate the *in situ* hybridization signal. The *DGW1* sense probe was used as a negative control (h). Bars = 200 μm. (j) Subcellular localization of DGW1 in *N*. *benthamiana* leaf cells (top) and rice protoplasts (bottom). OsMYB80‐RFP was used as a nuclear marker. Bars = 50 μm in top and 10 μm in bottom.

Quantitative real‐time PCR (RT‐qPCR) analysis revealed that *DGW1* exhibited ubiquitous expression across various rice tissues, including leaves, leaf sheaths, stems, roots and young panicles, with the highest expression level in developing panicles (Figure 3a,b). Accordingly, β‐glucuronidase (GUS) staining showed that the GUS signals of the transgenic rice plants expressing the *DGW1*‐promoter‐driven GUS reporter gene (*proDGW1:GUS*) were observed in stems, leaves, roots and preferentially in developing panicles (Figure 3c,d). Interestingly, we found that *DGW1* was highly expressed in the early stage of panicle development but gradually decreased as panicles matured (Figure 3b,d). Further, *in situ* hybridization analysis revealed that *DGW1* mRNA was abundantly expressed in the primordia of primary branches, secondary branches and young spikelets during panicle development (Figure 3e–i). These findings indicated that *DGW1* plays a pivotal role in rice panicle development.

We assessed the subcellular localization of DGW1 by translational fusion of the DGW1 to Yellow Fluorescent Protein (YFP). The transient expression of *35S:DGW1‐YFP* in both *N. benthamiana* leaf cells and rice protoplasts showed that DGW1‐YFP was exclusively localized to the nucleus, which was completely coincident with the signals of a nuclear marker OsMYB80‐RFP (Lei *et al*., 2022; Figure 3j). Therefore, we conclude that DGW1 is localized to and functions in the nucleus.

### Overexpression of *DGW1* results in large grains and increased grain yield

To further investigate the role of *DGW1* in regulating grain size, we generated the.

**Figure 4:**
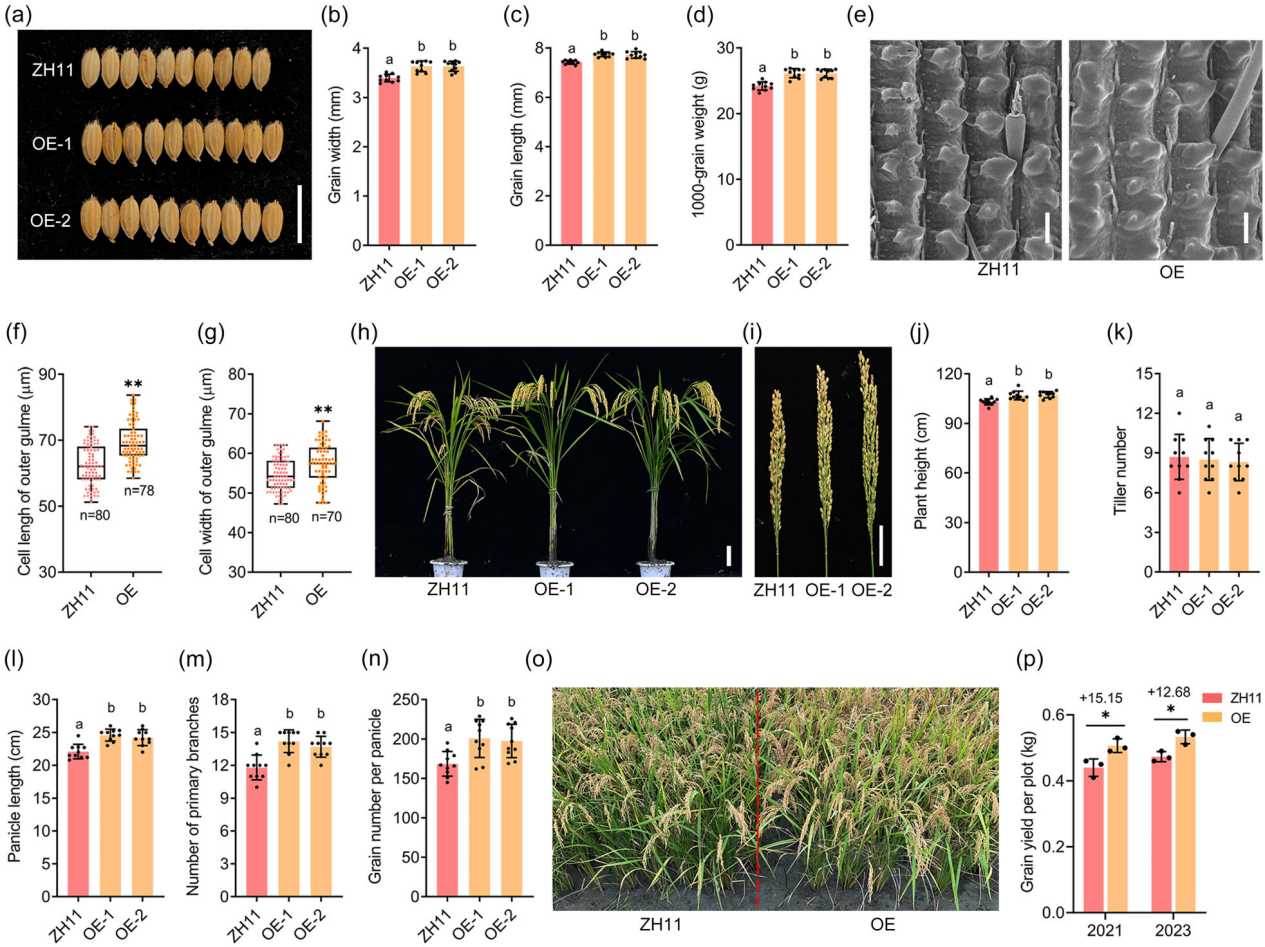
Overexpression of *DGW1* results in larger grain size and higher yield. (a) Mature paddy grains of ZH11, OE‐1 and OE‐2. Bar = 1 cm. (b‐d) Comparison of grain width (b), grain length (c) and 1000‐grain weight (d) of ZH11, OE‐1 and OE‐2. Values are means ± SD (*n* = 10). (e) SEM analysis of the outer surfaces of ZH11 and OE glumes. Bars = 100 μm. (f, g) Outer epidermal cell length (f) and cell width (g) of ZH11 and OE glumes. Values are means ± SD (*n* ≥ 70). (h, i) Mature plants (h) and panicles (i) of ZH11 and OE lines. Bars = 10 cm in (h), 5 cm in (i). (j‐n) Comparison between ZH11 and OE lines for plant height (j), tiller number (k), panicle length (l), number of primary branches (m) and grain number per panicle (n). Values are means ± SD (*n* = 10). (o) Phenotypes of ZH11 and OE plants grown in Chengdu, Sichuan, China in 2021. (p) Comparison of grain yield of ZH11 and OE in 2021 and 2023. Values are means ± SD (*n* = 3 plots, 20 plants within a plot). *P* value was determined using a *t‐*test compared with ZH11. *P < 0.05, **P < 0.01. Different lowercase letters indicate significant differences (*P* < 0.05).

*DGW1‐*overexpressing (OE) plants in the ZH11 background by fusing *DGW1* with 3 × FLAG tag. We obtained two stable transgenic lines exhibiting high levels of *DGW1* mRNA and protein expression (Figure S7). Compared to the ZH11, OE plants displayed a significant increase in grain size and weight (Figure 4a–d). Given the large grains produced by OE lines, we investigated whether overexpression of *DGW1* could promote cell expansion in spikelet hulls. The SEM observation showed that the outer glume cells were larger in OE lines than those of ZH11 (Figure 4e–g). These results supported that *DGW1* positively regulates rice grain size by influencing cell expansion in spikelet hulls. In terms of plant architecture and panicle morphology, OE lines exhibited significant increases in plant height, panicle length, primary branch number and grain number per panicle compared to ZH11 (Figure 4h–n). Field tests conducted in Chengdu in 2021 and 2023 demonstrated that overexpression of *DGW1* resulted in 15.15% and 12.68% increase in grain yield relative to ZH11 (Figure 4o,p). We speculated that the elevated grain yield of OE plants was attributed to a simultaneous enhancement of both grain number per panicle and grain weight.

### DGW1 binds to *GW6* mRNA

**Figure 5:**
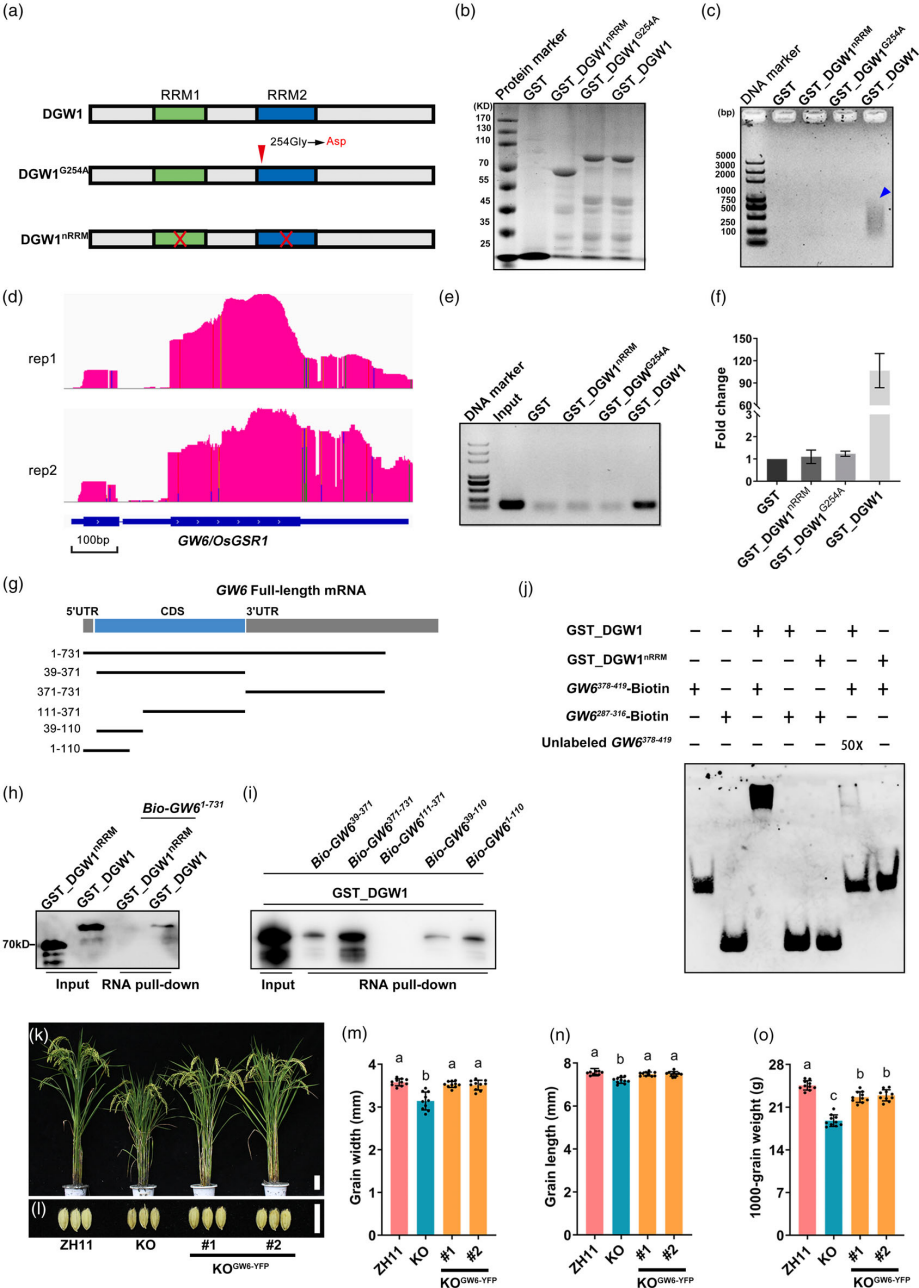
DGW1 binds to the *GW6* transcript. (a) Schematic structure of DGW1, DGW1^G254A^, DGW1^nRRM^. (b) Coomassie Blue‐stained SDS‐PAGE gel containing electrophoretically resolved GST fusion protein. (c) Agarose gel was used to detect RNA fragments bound by GST_DGW1^nRRM^, GST_DGW1^G254A^, GST_DGW1. GST was used as a control. The blue arrow shows the position of the RNA fragments. (d) Integrated Genome Browser (IGB) view tracks displaying the distributions of *GW6* transcript reads in PBR‐seq. (e, f) The binding of GST, GST_DGW1^nRRM^, GST_DGW1^G254A^ and GST_DGW1 to *GW6* transcripts was detected by RT‐PCR (e) and RT‐qPCR (f). (g) Schematic diagram of *GW6* mRNA fragments for RNA pull‐down assay. (h, i) Biotin‐labelled *GW6* mRNA fragments were tested for GST_DGW1 binding *in vitro*. GST_DGW1^nRRM^ protein was used as negative controls. (j) Competitive RNA‐EMSA experiment shows DGW1 binds to the U‐rich sequence of *GW6* mRNA. Unlabeled *GW6* ^*378‐419*^ probes were added into the reaction mixture as the competitive probes. GST_DGW1^nRRM^ was used as a control. (k, l) Plants and grains of ZH11, KO and two KO^*GW6‐YFP*^ lines. Bars = 10 cm in (k) and 1 cm in (l). (m‐o) Comparison of grain length (m), grain width (n) and 1000‐grain weight (o) of ZH11, KO and two KO^*GW6‐YFP*^ lines. Values are means ± SD (*n* = 10). Different lowercase letters indicate significant differences between ZH11 and other lines (Student's *t*‐test, *P* < 0.05).

DGW1 encodes an RBP that contains two classical RRM domains. To assess its RNA‐binding ability, we performed an *in vitro* protein binding RNA assay (PBR) using three recombinant fusion proteins: GST_DGW1, GST_DGW1^G254A^ (a *dgw1* mutant protein) and GST_DGW1^nRRM^ (a deletion mutant protein lacking two RRM domains; Figure 5a,b). Total rice RNA was incubated with these proteins while GST protein served as a negative control. RNA electrophoresis analysis showed that full‐length GST_DGW1 protein exhibited robust RNA binding activity *in vitro*, whereas both GST_DGW1^nRRM^ and GST_DGW1^G254A^ displayed negligible RNA binding activity (Figure 5c). These findings indicated that the RNA binding function of DGW1 was dependent on its RRMs and that the G254A mutation in *dgw1* impaired the RNA binding activity of DGW1.

To further identify mRNA targets that are regulated by DGW1, we performed high‐throughput sequencing of GST_DGW1‐enriched RNA in two independent PBR experiments. We identified 1278 and 2845 transcripts from the two experiments, respectively. Among them, 1152 transcripts were found in both experiments (Table S2). Subsequently, we increased the stringency and selected DGW1‐bound transcripts with high enrichment levels (Peak Fold Enrichment >10). As a result, a total of 139 transcripts were obtained and defined as putative target RNAs of DGW1 (Table S3). Notably, among these highly fold enrichment genes, *GW6* showed significantly higher binding enrichment levels (Table S3; Figure 5d), which belongs to the GAST family and is already known to positively regulate rice grain size (Shi *et al*., 2020). The results of reverse transcription‐PCR (RT‐PCR) and RT‐qPCR results showed that the mRNA abundance of *GW6* was only enriched in full‐length DGW1 protein, but not in GST, DGW1^nRRM^ or DGW1^G254A^ (Figure 5e,f). To further confirm the direct association between DGW1 and *GW6* mRNA, we conducted an *in vitro* RNA pull‐down assay utilizing biotinylated *GW6* mRNA (*Bio‐GW6* ^*1‐731*^; Figure 5g). We found that *Bio‐GW6* ^*1‐731*^ was capable of pulling down GST_DGW1, but not GST_DGW1^nRRM^ (Figure 5h). To gain a better understanding of the specific binding details between DGW1 and *GW6* mRNA, we designed five biotinylated RNA fragments covering different regions of the *GW6* mRNA sequence for RNA pull‐down experiments. Our results indicate that the 3’ UTR region of *GW6* (*Bio‐GW6* ^*371‐731*^) exhibited the strongest affinity for DGW1 *in vitro* (Figure 5g,i). To validate this observation, we then performed an RNA electrophoretic mobility shift assay (RNA‐EMSA). We synthesized two biotin‐labelled RNA probes: one containing a U‐rich sequence in the 3’UTR (*GW6* ^*378‐419*^‐Biotin), and another containing a sequence in the CDS (*GW6* ^*287‐316*^‐Biotin). We observed that only the migration of *GW6* ^*378‐419*^‐Biotin probe was retarded by GST‐DGW1, but not by the RRMs deletion variant GST_DGW1^nRRM^. Moreover, this migration was abolished upon the addition of unlabeled probe (Unlabeled *GW6* ^*378‐419*^; Figure 5j). These results strongly suggest that DGW1 directly binds to *GW6* mRNA, particularly in the *GW6* 3’UTR region. To investigate the genetic correlation between *DGW1* and *GW6*, we conducted an overexpression experiment of *GW6* in *DGW1* KO plants. The results demonstrated that the grain size of KO lines was effectively restored (Figure 5k–o), indicating that *GW6* may act genetically downstream of *DGW1* to regulate rice grain size. These findings suggest that the *GW6* mRNA is the main target of DGW1 in rice grain size regulation.

Since RBPs play a crucial role in various aspects of mRNA metabolism, including transcriptional and post‐transcriptional processes (Lorkovic, 2009), we were prompted to investigate how DGW1 regulates *GW6* function by binding to its mRNA. The RT‐qPCR results showed that the mRNA level of *GW6* in *dgw1* panicles was slightly decreased compared to that of the wild‐type (Figure S8a). To assess the turnover rates of *GW6* mRNA in wild‐type and *dgw1*, we designed two primers targeting the 5′‐ and 3′‐ ends of *GW6* mRNA respectively for RT‐qPCR assay. The ratio of 5′‐ to 3′‐ ends of *GW6* mRNA showed no significant difference between the wild‐type and *dgw1*, indicating that DGW1 did not alter the turnover rates of *GW6* mRNA (Figure S8b). We also examined the splicing efficiency of *GW6* by calculating the expression levels of exons relative to their corresponding introns. The RT‐qPCR analysis showed that there was no significant difference in the splicing efficiency of *GW6* intron between *dgw1* and wild‐type (Figure S8c). We next hypothesized that DGW1 might influence the translational level of *GW6*. Since mRNA is translated on the ribosome, we extracted polyribosomes from young panicle of wild‐type and *dgw1* by sucrose centrifugation and isolated the mRNA being translated. RT‐qPCR analysis showed that the polyribosomes‐bound *GW6* mRNA of *dgw1* was significantly decreased compared with that of wild‐type (Figure S9c). Further transient co‐expression assays in *N. benthamiana* epidermal cells showed that the co‐expression of DGW1‐mCherry led to an increase in GW6‐YFP protein abundance and a deceleration in protein decay, compared to co‐expression with mCherry protein (Figure S9a,b). Overall, our results suggest that the interaction between DGW1 and *GW6* mRNA may have an impact on GW6 protein levels.

### DGW1 participates in the response of GA and BR in rice

**Figure 6:**
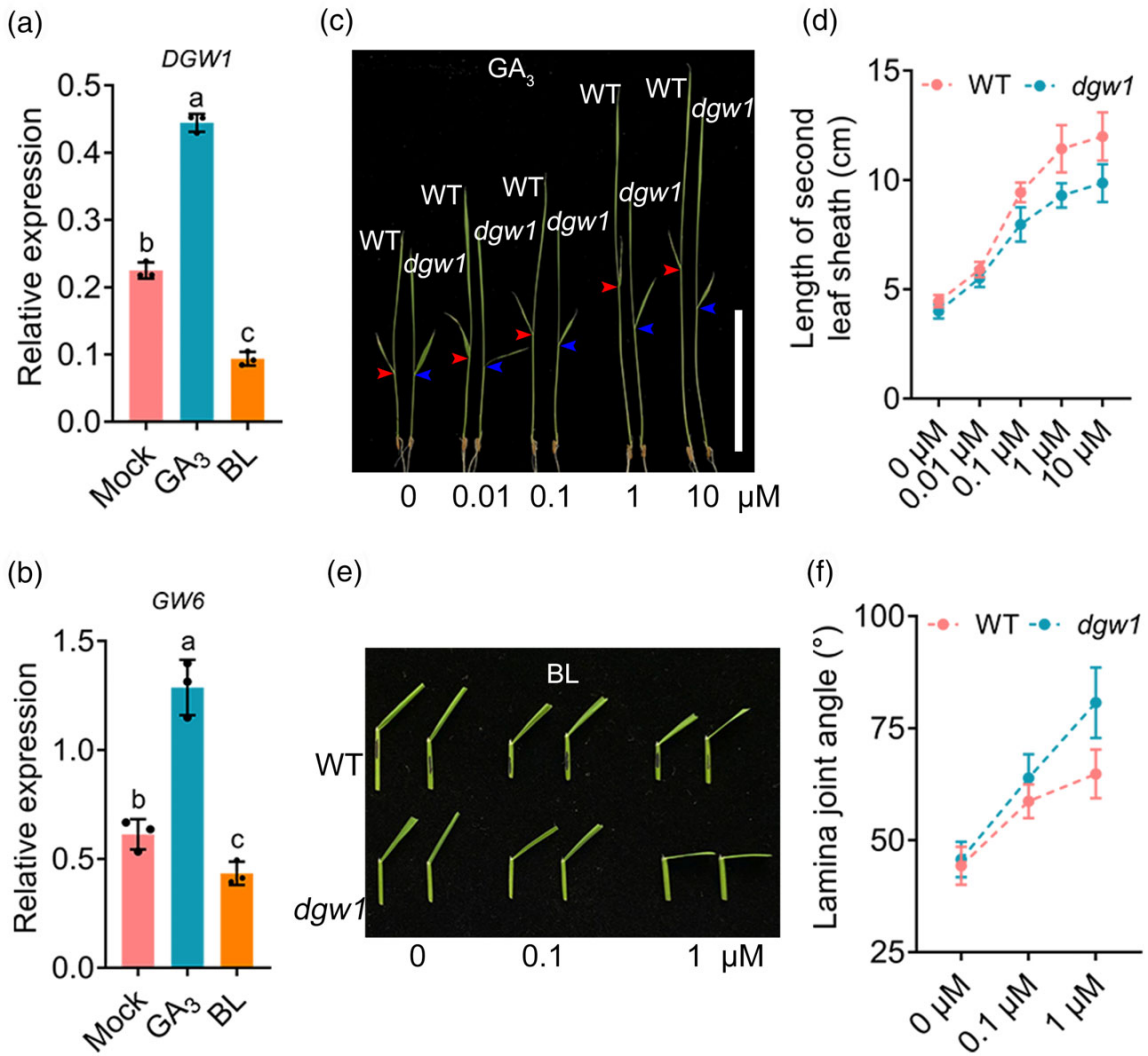
*DGW1* responds to GA and BR. (a, b) Expression levels of *DGW1* (a) and *GW6* (b) in 14‐day‐old WT seedlings upon 10 μM GA_3_ or 1 μM 24‐epibrassinolide treatment. Data are means ± SD (*n* = 3). Different lowercase letters indicate significant differences (*P* < 0.05). (c) Dose‐dependent responses of WT and *dgw1* seedlings to GA_3_ treatment. The red and blue arrows indicate the second leaf of WT and *dgw1*, respectively. Bar = 10 cm. (d) Comparison of second leaf sheath length between WT and *dgw1* with GA_3_ treatment. Data are means ± SD (*n* = 20). (e) The leaf inclination of WT and *dgw1* in the presence of indicated concentration of 24‐epibrassinolide. (f) Statistical analysis of leaf inclination in (e). Values are means ± SD (*n* = 20).

Previous studies have demonstrated that *GW6* integrates GA and BR signalling to regulate grain size and plant architecture (Li *et al*., 2021; Shi *et al*., 2020; Tang *et al*., 2021; Wang *et al*., 2009). Therefore, we sought to investigate whether *DGW1* and *GW6* function together in a common regulatory pathway. It has been reported that GA induces the expression of *GW6* while BR inhibits it (Wang *et al*., 2009). We first examined the expression levels of *DGW1* and *GW6* after treatment with GA_3_ and 24‐epibrassinolide (BL). Our results showed that these two genes were induced by GA_3_ treatment but inhibited by BL treatment (Figure 6a,b), indicating that *GW6* and *DGW1* exhibit similar expression patterns in response to GA and BR. Moreover, we assessed the sensitivity of seedlings to GA_3_ in both wild‐type and *dgw1* by measuring the length of the second leaf sheath. As expected, GA_3_ promoted the elongation of the second sheath, which was reduced in *dgw1* and KO lines compared to the wild‐type and ZH11, respectively (Figure 6c,d and Figure S10a,b). In addition, we performed RNA‐seq analysis using ZH11 and KO seedlings and found that several downregulated genes in KO were associated with the GA synthesis and signalling, such as *ent‐kaurene oxidases 5* (*OsKO5*) (Itoh *et al*., 2004), *Growth Regulating Factors* (*OsGRF1*, *OsGRF6* and *OsGRF7*) (Chen *et al*., 2020; Tang *et al*., 2018; van der Knaap *et al*., 2000), *GA‐stimulated transcript‐related gene 2* (*OsGASR2*) (Furukawa *et al*., 2006; Table S1; Figure S11a). The mature *dgw1* plants exhibit typical BR‐deficient phenotypes such as erect leaves, reduced plant height and compact architecture (Figure S1). The lamina joint inclination assay showed that both *dgw1* and KO lines were more responsive to treatments with 0.1 and 1 μM BL, exhibiting greater bending of lamina joints than the wild‐type and ZH11, respectively (Figure 6e,f; Figure S10c,d). Moreover, RNA‐seq analysis showed that some BR synthesis and signalling genes were down‐regulated in KO exemplified by *XIAO* (Jiang *et al*., 2012), *dwarf and low‐tillering* (*DLT*) (Tong *et al*., 2012), *slender grain Dominant* (*SLG*) (Feng *et al*., 2016), *CYP734A4* (Sakamoto *et al*., 2011; Table S1; Figure S11b). These findings demonstrate that *DGW1* and *GW6* exhibit a similar expression pattern in response to GA and BR signalling. Moreover, *dgw1* displayed reduced response to GA but increased response to BR, just like the *GW6*‐deficient mutant (Shi *et al*., 2020; Wang *et al*., 2009), indicating that *DGW1* and *GW6* may function in a common pathway regulating multiple aspects of rice growth.

### DGW1 interacts with OsUBP1s to binding *GW6* RNA

**Figure 7:**
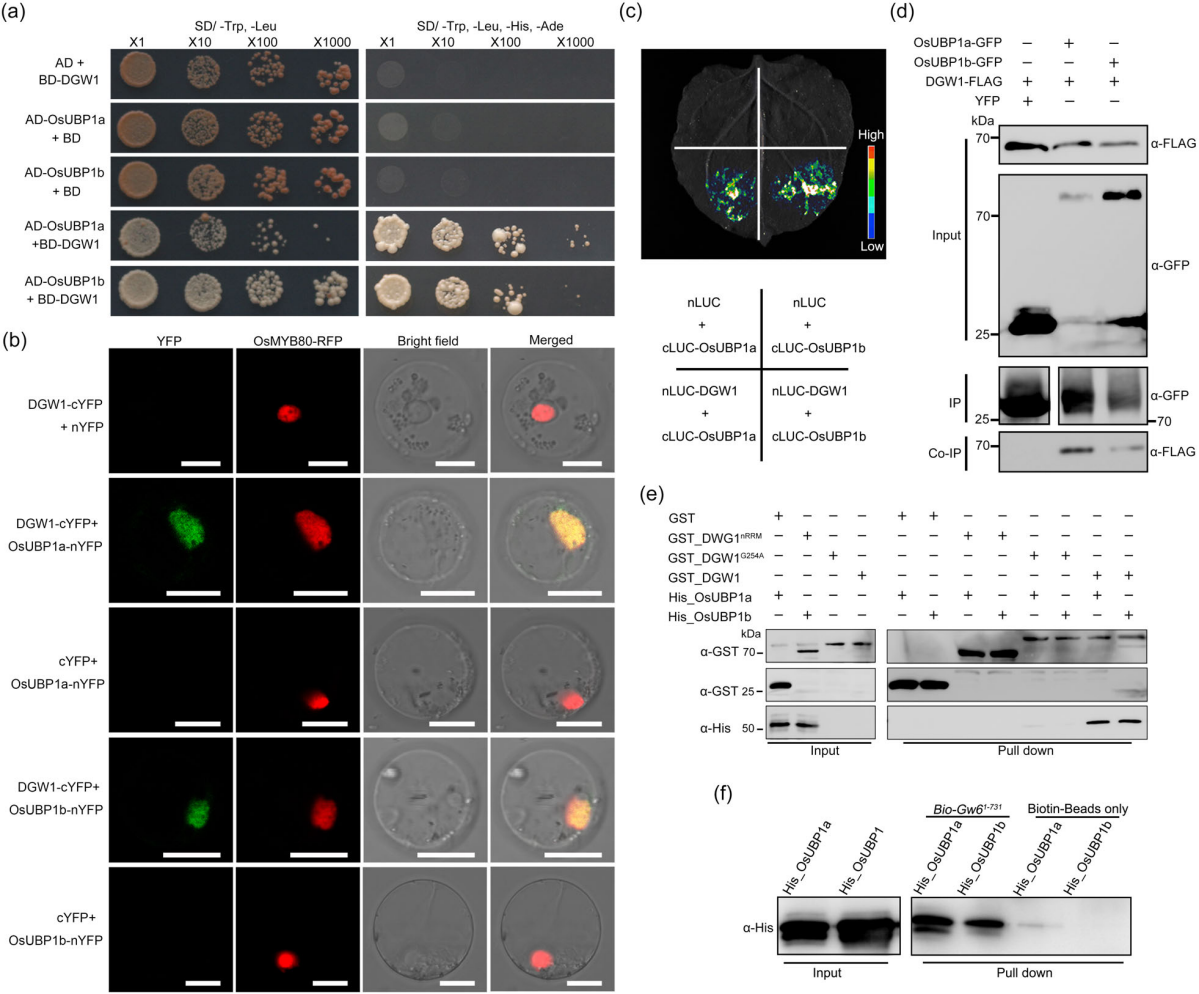
DGW1 physically interacts with OsUBP1a/b *in vitro* and *in vivo*. (a) Yeast two‐hybrid assays indicate that DGW1 interacts with OsUBP1a/b. The yeast cells were cultured on SD/−Trp‐Leu (medium without tryptophan and leucine) or SD/−Trp‐Leu‐His‐Ade (medium without tryptophan, leucine, histidine and adenine). (b) DGW1 associates with OsUBP1a/b, as shown by BiFC assays in rice protoplast. DGW1 was fused to the C‐terminal fragment of YFP (cYFP) and OsUBP1a/b was fused to the N‐terminal fragment of YFP (nYFP). OsMYB80‐RFP was used as a nuclear marker. Bars = 10 μm. (c) LCI assay indicates that DGW1 interacts with OsUBP1a/b in *N. benthamiana* leaves. Co‐transformation of nLUC‐DGW1 and cLUC‐OsUBP1a/b led to the reconstitution of LUC signal, whereas no signal was detected when nLUC and cLUC‐ OsUBP1a/b were co‐expressed. In each experiment, at least three independent *N. benthamiana* leaves were infiltrated and analysed. (d) Co‐immunoprecipitation assays indicated that DGW1 interacts with OsUBP1a/b. *DGW1‐FLAG* and *OsUBP1a/b‐GFP* fusions were co‐expressed in *N. benthamiana* leaves. Proteins were immunoprecipitated (IP) with GFP beads and detected with anti‐GFP (α‐GFP) antibody. Co‐immunoprecipitated (Co‐IP) proteins were probed with anti‐FLAG (α‐FLAG) antibody. (e) Pull‐down assay indicated that DGW1 interacts with OsUBP1a/b. GST_DGW1^nRRM^, GST_DGW1^G254A^, GST_DGW1 was incubated with His_OsUBP1a/b and pulled down by GST beads and detected by immunoblot with anti‐His antibody. GST were used as controls. (f) Biotin‐labelled *GW6* mRNA fragments were tested for OsUBP1a/b binding *in vitro*. Biotin beads were used as a negative control.

Previous studies have demonstrated that UBA2a and UBA2b, which belong to the UBA family, interact with AtUBP1b to contribute to mRNAs stabilization within the nucleus (Lambermon *et al*., 2000, 2002). We identified three homologous proteins of AtUBP1b in rice, which we designated as OsUBP1a, OsUBP1b and OsUBP1c, respectively (Figure S12). We selected OsUBP1a and OsUBP1b for further protein interaction analysis. The results of yeast two‐hybrid assays indicated that DGW1 interacts with OsUBP1a/b (Figure 7a). The bimolecular fluorescence complementation (BiFC) assays in rice protoplast displayed robust YFP fluorescence signals overlapping with the nuclear marker OsMYB80‐RFP upon co‐transfection of DGW1‐cYFP and OsUBP1a/b‐nYFP (Figure 7b). Our co‐localization assays showed that OsUBP1a/b‐YFP and DGW1‐mCherry co‐localized with the nuclear marker OsMYB80‐CFP in the nucleus, providing compelling evidence for a plausible interaction (Figure S13). Consistently, Luciferase Complementation Imaging (LCI) assay revealed detectable luciferase signals upon co‐expression of nLUC‐DGW1 and cLUC‐OsUBP1a/b (Figure 7c). The co‐immunoprecipitation (Co‐IP) assay showed that OsUBP1a/b‐GFP proteins co‐immunoprecipitated with the DGW1‐FLAG protein (Figure 7d). In addition, *in vitro* pull‐down assays showed that GST_DGW1 could pull down His_OsUBP1a/b, while GST, GST_DGW1^nRRM^ or GST_DGW1^G254A^ could not, suggesting the interactions were dependent on the RRM domains of DGW1 (Figure 7e). In summary, these *in vivo* and *in vitro* data demonstrated that DGW1 physically interacts with OsUBP1a/b.

Considering that OsUBP1a/b also encode RBPs with RRM domains, we speculated that they might bind the same RNA target as DGW1. Therefore, we performed an RNA pull‐down assay to detect whether OsUBP1a/b was able to bind *GW6* mRNA. The results showed that biotinylated *GW6* RNA indeed pulled down the His_OsUBP1a/b protein *in vitro* (Figure 7f). These results suggested that DGW1 interacts with OsUBP1a/b and may form complexes of RBPs to target *GW6* mRNA.

## Discussion

Post‐transcriptional regulation of RNA metabolism is increasingly recognized as a pivotal step in the precise control of gene expression (Hentze *et al*., 2018). RBPs play a central role in regulating post‐transcriptional RNA metabolism throughout plant growth, development and stress responses (Lee and Kang, 2016; Nguyen *et al*., 2016). The genetic and molecular mechanisms underlying post‐transcriptional regulation of grain size in rice by RBPs are not yet fully understood. In this study, we identified an hnRNP‐like RBP, DGW1, which acts as an essential post‐transcriptional regulator that directly binds to *GW6* mRNA to positively regulate grain size in rice.

*DGW1* encodes an hnRNP‐like RBP with two RRM domains (Figure S6a). RBPs interact with target RNAs to form ribonucleoprotein complexes that govern the post‐transcriptional processing of RNA, thereby finely tuning gene expression regulation and controlling diverse physiological processes during plant growth and development (Yeap *et al*., 2014). In this study, we demonstrate that a loss‐of‐function mutation in *DGW1* results in pleiotropic defects of grain size and plant architecture (Figure 1; Figure S1). An interesting question is whether these traits are all regulated through the same direct mRNA target of DGW1, or whether there are multiple mRNA targets of DGW1. A recent study had revealed that *DHT1*, the same gene locus as *DGW1*, which was cloned from a mutant exhibiting dwarfism and increased tiller numbers, functions as a splicing component and inhibits rice tillering by regulating appropriate pre‐mRNA splicing of the SL receptor gene *D14* (Liu *et al*., 2022c). Overexpression of *D14* in the *dht1* mutant effectively rescued its tiller number phenotype, indicating that *D14* mRNA is the target of DHT1/DGW1 in rice tillering regulation (Liu *et al*., 2022c). In our study, through various protein‐RNA interactions and genetic assays, we demonstrated that *GW6* mRNA was the main target of DGW1 in regulating rice grain size. *GW6* encodes a GA‐regulated GAST family protein and coordinates BR and GA signalling to positively regulate grain size in rice (Shi *et al*., 2020; Tang *et al*., 2021; Wang *et al*., 2009). *DGW1* and *GW6* loss of function mutants exhibited identical phenotypes of the smaller size of grain and panicle (Figure 2c–f; Figure S3; Shi *et al*., 2020; Tang *et al*., 2021; Wang *et al*., 2009). We found that *DGW1* and *GW6* have similar expression patterns, such as high expression in young panicles and meristems, induced by GA and inhibited by BR (Figure 3a–d and Figure 6a,b; Shi *et al*., 2020; Tang *et al*., 2021; Wang *et al*., 2009). The *dgw1* mutant was sensitive to BR and insensitive to GA (Figure 6c–f), which was consistent with the *GW6* deficient mutant (Shi *et al*., 2020; Wang *et al*., 2009). The overexpression of *GW6* in *DGW1*‐KO plants effectively restored the grain size phenotype (Figure 5k–o). These results provided multiple lines of evidence that *GW6* transcript is a direct downstream target of DGW1 in grain size controlling. Additionally, it implies that *DGW1/DHT1* may have diverse roles in regulating rice growth and development through its interaction with mRNA from various target genes.

HnRNPs represent a large family of RBPs that contribute to multiple aspects of RNA metabolic processes, including pre‐mRNA splicing, mRNA stability, localization and translation control (Bove *et al*., 2008). UBA2s, the UBA proteins in *Arabidopsis*, have been reported to interact with hnRNP protein UBP1b. The UBP1b and UB2As are constituents of complexes that recognize U‐rich sequences in plant 3’UTR and contribute to mRNA stabilization in the nucleus (Lambermon *et al*., 2002). In higher plants, UBP1 specifically binds to U‐rich sequences to mediate post‐transcriptional regulation of genes and is widely involved in plant growth, development and stress response (Lambermon *et al*., 2000, 2002; Nguyen *et al*., 2016; Sorenson and Bailey‐Serres, 2014). In this study, we demonstrated that DGW1 physically interacts with OsUBP1a and OsUBP1b, and all three hnRNP‐like proteins bind to the same mRNA targets, *GW6* (Figure 7). Previous studies have demonstrated that the RRM domains of RBPs are required for their protein‐RNA and protein–protein interactions (Maris *et al*., 2005; Tian *et al*., 2018). Consistently, our results showed that DGW1^nRRM^ and DGW1^G254A^ mutant proteins not only lost their RNA binding ability but also disrupted their interaction with OsUBP1s (Figure 5c, e, f and Figure 7e), indicating that the protein‐RNA and protein–protein interaction mediated by DGW1 are dependent on the RRM domains. Based on these findings, we propose that the DGW1‐OsUBP1a/b may function as an hnRNP complex to regulate the fate of *GW6* mRNA. In the current study, we found no significant differences in the mRNA turnover rates and pre‐mRNA splicing efficiency of *GW6* between the *dgw1* mutant and the wild‐type (Figure S8). Moreover, we found polyribosomes‐bound *GW6* mRNA of *dgw1* was significantly decreased compared with that of wild‐type (Figure S9c). Further transient co‐expression assays suggested that the presence of DGW1 led to an increase in cellular accumulation and stability of GW6 protein (Figure S9a,b). Thus, we speculate that DGW1‐OsUBP1a/b may bind to *GW6* mRNA to regulate its translation level in rice. Further studies are needed to decipher how DGW1 and OsUBP1a/b fine‐tune the post‐transcriptional and translational regulation of *GW6*.

Fine manipulation of grain size effectively improves rice yield and quality (Zhao *et al*., 2018). The positive regulators of grain size and weight possess significant potential for enhancing yield in modern rice breeding. For instance, upregulating the expression of *GRAIN SIZE ON CHROMOSOME 2* (*GS2*) results in larger and more abundant cells, thereby enhancing grain weight and yield (Hu *et al*., 2015). Overexpressing *GRAIN WEIGHT 8* (*GW8*) stimulates cell division and grain filling, leading to increased grain width and yield (Wang *et al*., 2012). The elevated expression of *Grain weight 6a* (*GW6a*) promotes an increase in grain weight and yield by enhancing the number of spikelet cells and improving grain filling (Song *et al*., 2015). The grain size is determined by cell differentiation, as well as either cell proliferation or expansion during panicle development (Guo *et al*., 2018). In the present study, we observed a high expression level of *DGW1* during rice panicle development, particularly in the primordia of primary and secondary branches as well as young spikelets (Figure 3e–i). The cytological observations indicate that overexpression of *DGW1* in rice leads to a significant increase in cell expansion, resulting in larger grain size and weight (Figure 4a–g). Remarkably, the field tests demonstrated that *DGW1‐OE* plants exhibited a significant increase in grain weight and grain number per panicle, two crucial components of rice yield, which eventually led to a substantial enhancement of rice grain yield (Figure 4h–p). Therefore, our findings provide a potential strategy for increasing rice yield by manipulating the expression of *DGW1*.

## Experimental procedures

### Plant material and culture conditions

The *dgw1* mutant was isolated from ethyl methanesulfonate‐treated seeds of the *indica* rice cultivar, shuhui600 (R600). All plants were grown in experimental fields of Sichuan Agricultural University in Chengdu, Sichuan, China, under natural conditions.

### Morphological and histological analysis

The grain size of all plants was measured using the SC Detection and Analysis System (WSeen, China).

For resin sections, spikelet hulls and culms were fixed in FAA solution (50% ethanol, 5% acetic acid and 3.7% formaldehyde) for 24 h after 30 min vacuum treatment. Tissues were embedded in resin (Technovit 7100, Germany). Sections (5 μm) were prepared with a microtome (RM2255, Leica) and observed with a microscope (AxioScope A1, Zeiss).

For SEM analysis, spikelet hulls were fixed in 1× PBS solution 24 h at 4 °C that contained 2.5% glutaraldehyde after 30 min vacuum treatment and dehydrated in a graded alcohol series. Cells of the middle part of the lemma were observed and imaged with an SEM (SU3500, Hitachi).

### Molecular cloning of *DGW1*

To map the *DGW1*, we generated backcross F_2_ segregation population by crossing wild‐type and *dgw1*. The DNA of 40 individuals with smaller grains of the F_2_ population were pooled, and next‐generation sequencing was performed (Novogene, China). The MutMap approach was utilized to identify mutations linked with the *dgw1* phenotype (Abe *et al*., 2012).

### Vector construction and plant transformation

For complementation, a 6665‐bp genomic fragment that contained the *DGW1* (*LOC_Os04g54440*) genomic sequence, coupled with 2617‐bp upstream, was amplified from wild‐type using primers PHB‐54440FL. The fragment was inserted into the binary vector PHB digested with *Eco*RI and *Hin*dIII.

To generate DGW1‐overexpression lines, the full‐length coding sequence of *DGW1* was amplified from the cDNAs of Nipponbare using primers 2300‐DGW1OE. The fragment was inserted into the binary vector pCAMBIA2300‐FLAG under the control of the *ubiquitin* promoter.

To generate the *DGW1* knockout lines by the CRISPR/Cas9 system, a 20‐bp target sequence was selected for specific recognition of *DGW1*/*LOC_Os04g54440*. The fragment was cloned into the vector BGK03 (Biogle, China).

For promoter activity assay, a 2617‐bp *DGW1* promoter region from wild‐type was fused to the GUS reporter gene with the nopaline synthase terminator and inserted into the PHB‐GUS vector.

To generate the overexpression vector PHB‐YFP_*GW6*, the coding region of *GW6* was amplified and the PCR product was cloned into the binary vector PHB‐YFP under the control of the CaMV 35S promoter.

### RNA extraction and RT‐qPCR

Total RNA was isolated using a Plant Total RNA Isolation Kit (FOREGENE Co., Ltd, China), and 1 μg RNA was reverse‐transcribed by oligo (dT) primers using a HiScript II 1st Strand cDNA Synthesis Kit (Vazyme Biotech, China). RT‐qPCR was performed on qTOWER3G machines (Analytik Jena, Germany) using Taq Pro Universal SYBR qPCR Master Mix (Vazyme Biotech, China) according to the manufacturer's instructions. The *ACTIN* (*LOC_Os03g0718100*) gene was used as the internal control.

### GUS staining and RNA *in situ* hybridization

GUS activity was detected using GUS histochemical assay kit (Real‐Times, China) according to the manufacturer's manual. RNA *in situ* hybridization was carried out following the method described previously (Cheng *et al*., 2018), and images were captured using panoramic MIDI (3DHISTECH Ltd, Hungary).

### Subcellular localization

For subcellular localization analysis, the coding sequences of *DGW1* and *OsUBP1a/b* were cloned into the binary vectors PHB‐YFP or pCAMBIA2300‐mcherry under the control of the *CaMV 35S* promoter. These constructs were introduced into *Nicotiana benthamiana* leaf cells or rice protoplasts for transient expression following the methods described previously (Page *et al*., 2019; Sparkes *et al*., 2006). We selected the rice MYB transcription factor OsMYB80 as a nuclear marker, which is known for its consistent nuclear localization (Lei *et al*., 2022). The fluorescence of fusion protein was observed using a confocal laser scanning microscope (A1‐90i, Nikon).

### RNA‐sequencing analysis

Seedlings (12‐day‐old) of ZH11 and KO were collected and immediately frozen in liquid nitrogen. The extraction and examination of total RNA, library preparation and Illumina sequencing were done by Genedenovo Biotechnology (Guangzhou, China). The filtered clean reads were aligned to the rice Nipponbare reference genome and genes (IRGSP‐1.0) using TopHat2/ Bowtie2 software. Differential gene expression was determined with the DEseq2 using the criteria of fold‐change ≥2 and *q*‐value ≤ 0.05.

### Protein expression and purification

To express the GST fusion protein, the coding sequences of *DGW1*, *DGW1* ^*nRRM*^ and DGW1^G254A^ were inserted into the pGEX‐4 T‐1 vector. The CDS of *OsUBP1a* and *OsUBP1b* were cloned into the pET28a vector to express the His fusion protein. The recombinant pGEX‐4 T‐1 and pET28a vectors were then transformed into BL21‐competent (DE3) cells. Fusion proteins were induced with 0.25 mM IPTG at 28 °C for 4 h. Mag‐Beads GST Fusion Protein Purification kit (Sangon Biotech, China) and His‐tag Purification Resin kit (Beyotime, China) were used for fusion protein purification.

### Protein binding RNA assay

Protein binding RNA assay was performed as described previously with modifications (Ma *et al*., 2022; Tian *et al*., 2019b; Zhou *et al*., 2018). The total RNA extracted from various developmental stages of panicles (0–10 cm) of wild‐type was heated to 95 °C for 2 min, then slowly cooled down to room temperature for annealing. For binding of RNAs by DGW1 protein, the GST protein lysate was incubated with GST Mag‐beads (Sangon Biotech, China) washed three times with binding/washing buffer (140 mM NaCl, 2.7 mM KCl, 10 mM Na_2_HPO_4_, 1.8 mM KH_2_PO_4_, pH 7.4). Then, add total RNA to mixtures, samples were incubated in pull‐down buffer (6.5 mM sodium phosphate pH 7.4, 140 mM NaCl, 0.02% Tween 20 and 40 U/mL RNase inhibitor) for 3 h at 4 °C. The protein–RNA complexes were eluted with elution buffer (50 mM Tris–HCl, 10 mM Glutathione reduced, PH 8.0). The eluted samples were subjected to RNA extraction using Trizol (Beyotime, China), followed by sequencing (Novogene, China).

### RNA pull‐down assay

RNA pull‐down assay was prepared as described previously (Zhou *et al*., 2018). The RNA fragments were transcribed with T7 High Efficiency Transcription Kit (TransGen Biotech, China) and labelled with biotin using the Pierce RNA 3′ end Desthiobiotinylation Kit (Thermo Fisher Scientific, USA). The RNA‐protein pull‐down assay was performed by Pierce Magnetic RNA‐Protein Pull‐Down Kit (Thermo Fisher Scientific, USA). 50 pmol biotinylated RNAs were added to the magnetic beads and incubated for 30 min at room temperature with agitation. Add fusion protein to the magnetic beads, then incubate for 2 h at 4 °C. The beads were then washed five times with wash buffer (20 mM Tris at PH 7.5, 10 mM NaCl, 0.1% Tween‐20) and boiled in SDS buffer. Proteins were detected by western blot with appropriate antibodies, anti‐GST (Abmart, MA9024), anti‐His (Beyotime, AF2870).

### RNA EMSA assay

RNA oligoribonucleotide probes were synthesized and labelled with biotin at the 3′ end (Tsingke Biotech Co., Ltd, China). The RNA EMSA assay was carried out with the RNA‐EMSA/Gel‐shift Kit (BersinBio, China) according to the manufacturer's instructions. To determine the specificity of the RNA‐protein interaction, the unlabeled probes were added to the reaction mixture and the GST‐DGW1^nRRM^ protein was used as a negative control. RNA probe sequences are shown in Table S4.

### Isolation of polysomal mRNA

Polysome isolation from young panicles of wild‐type and *dgw1* was performed according to a previously described method (Mustroph *et al*., 2009). Polysome‐RNA was isolated using Trizol (Beyotime, China) according to the manufacturer's protocol. RT‐qPCR was performed as described previously.

### Yeast two‐hybrid assay

Yeast two‐hybrid assay was performed with the Y2H Gold‐Gal4 system (Clontech, USA). The CDS of *DGW1* was introduced into the pGBKT7 vector, and full‐length cDNA of *OsUBP1a* and *OsUBP1b* were cloned into the pGADT7 vector. The constructs were co‐transformed into Y2H Gold cells according to the manufacturer's instructions (Clontech, USA). Interactions were detected on the SD/−Trp‐Leu‐His‐Ade medium.

### BiFC assay

For the BiFC assays, the full‐length cDNA of *DGW1* was cloned into pEarleyGate 101 (c‐YFP), the CDS of *OsUBP1a* and *OsUBP1b* were cloned into pEarleyGate 104 (n‐YFP). The vectors were then co‐transformed into rice protoplasts for transient expression (Page *et al*., 2019). The fluorescence was visualized using a confocal laser scanning microscope (STELLARIS STED/EM CDP300, Leica).

### LCI assay

The coding sequences of *DGW1* and *OsUBP1a/b* were inserted into pCAMBIA‐nLUC and pCAMBIA‐cLUC, respectively. Different combinations of *Agrobacterium* GV3101 containing the recombinant plasmids were cotransformed into *N. benthamiana* leaves. Luciferase activity was measured using chemiluminescence imaging (ChemiScope 6200) after 36 h of transformation.

### Co‐IP assay

For Co‐IP assays, the full‐length coding sequences of *DGW1* and *OsUBP1a/b* were subcloned into pCAMBIA2300‐FLAG and pCAMBIA2300‐GFP vectors, respectively. The *DGW1*‐*FLAG* and *OsUBP1a/b*‐*GFP* constructs in *Agrobacterium* strain GV3101 were transiently co‐expressed in *N. benthamiana* leaves. Total proteins were extracted with protein extraction buffer (10% glycerol, 50 mM Tris–HCl at pH 7.5, 150 mM NaCl, 1 mM PMSF, 1 mM EDTA at pH 8.0, 1 mM DTT, 0.25% Triton X‐100, 0.25% NP‐40, 1× protease inhibitor cocktail). Anti‐GFP Nanobody magarose Beads (AlpalifeBio, China) were added to the protein supernatant, and incubated at 4°C for 3 h. Beads were washed three times with the washing buffer (50 mM Tris–HCl at pH 7.5, 150 mM NaCl, 1 mM EDTA at pH 8.0) and boiled in SDS loading buffer at 100°C for 8 min. The immunoprecipitates were analysed by SDS–PAGE and immunoblotted with anti‐GFP (Sangon Biotech, D191040) and anti‐FLAG (Sigma, F1804) antibodies.

### Pull‐down assay

Pull‐down assay was prepared as previously described (He *et al*., 2021). The recombinant GST fusion proteins were added to the Glutathione Mag‐Beads (Sangon Biotech, China) for 2 h at 4 °C with agitation and then washed with binding/washing buffer three times. The recombinant His‐OsUBP1a/b proteins were then added to the beads complex and incubated for another 2 h at 4 °C. After five times of beads washing, the complexes were eluted with an elution buffer. Proteins were detected by western blot with appropriate antibodies, anti‐GST (Abmart, MA9024), anti‐His (Beyotime, AF2870).

### Lamina joint assay

The lamina joint assay was performed as described previously with modifications (Li *et al*., 2021). The second leaf laminas of 10‐day‐old seedlings were submerged in the solutions containing different 24‐epibrassinolide concentrations for 24 h in the dark. The angles of lamina joint bending were measured using ImageJ software.

### Gibberellin‐induced elongation of the second leaf sheath

The germinated seeds of wild‐type, ZH11, *dgw1* and KO were grown on 96‐well plates in Yoshida rice seedling culture solution with a gradient concentration of GA_3_ under 12‐h light/12‐h dark conditions. After 8 days, the length of the second leaf sheath was measured.

### Statistical analysis

All bar chart plots and line charts were processed and presented using GraphPad Prism 9. The statistical analysis by the two‐tailed, two‐sample *t*‐test was performed using SPSS Statistics 27.

### Accession numbers

The RNA‐seq and PBR‐seq data of this article have been deposited in the National Center for Biotechnology Information Sequence Read Archive under accession numbers PRJNA901911 and PRJNA902119, respectively.

Sequence from this study can be downloaded from the rice genome annotation project (http://rice.plantbiology.msu.edu/) with the following accession numbers:, LOC_Os04g54440 (DGW1), LOC_Os06g15620 (GW6), LOC_Os11g40510 (OsUBP1a), LOC_Os08g40880 (OsUBP1b), LOC_Os07g42380 (OsUBP1c), LOC_Os06g02019 (OsKAO), LOC_Os06g37224 (OsKO5), LOC_Os12g29980 (OsGRF7), LOC_Os04g39110 (OsGASR2), LOC_Os02g53690 (OsGRF1), LOC_Os03g51970 (OsGRF6), LOC_Os04g48760 (XIAO), LOC_Os06g03710 (DLT), LOC_Os08g44840 (SLG), LOC_Os03g07540 (OsbHLH153), LOC_Os06g39880 (OsCYP734A4), LOC_Os04g56990 (RLI1), LOC_Os01g50410 (OsMKKK70), LOC_Os03g50885 (ACTIN).

## Author contributions

Y Liang and P Li designed the research; LL performed the experiments with assistance from JL, KL, WJ, JY, TQ, CJ, BL, ZC, JL, FL, XL, PW, JJ, QD, SW, JZ, TZ, HL, SL; LL, Y Liang and P Li analysed the data; Y Liang and LL wrote the manuscript with improvement from P Li.

## Conflict of interest statement

The authors declare no conflict of interest in this study.

## Supporting information


**Figure S1** Other phenotypes of *dgw1* mutant.
**Figure S2** Histocytological analysis of stem of WT and *dgw1*.
**Figure S3** Plant and panicle phenotypes of the *DGW1*‐CRISPR/Cas9 knockout plants.
**Figure S4** Histological analysis of the *DGW1*‐CRISPR knockout plant.
**Figure S5** Plant and panicle phenotypes of complemented transgenic lines.
**Figure S6** Bioinformatics analysis of DGW1 protein.
**Figure S7** mRNA and protein levels of *DGW1* in OE lines.
**Figure S8** mRNA level and pre‐mRNA splicing efficiency of *GW6* in WT and *dgw1*.
**Figure S9** Transient co‐expression of *DGW1* and *GW6* and polyribosomes‐bound *GW6* mRNA analysis.
**Figure S10** Response of KO plants to BR and GA treatments.
**Figure S11** Heat map representing the transcript abundance of GA‐ and BR‐related differentially expressed genes in RNA‐seq.
**Figure S12** Multiple sequence alignment of AtUBP1b with its homologues in rice.
**Figure S13** Co‐localization of DGW1 with OsUBP1a/b.

**Table S1** Differentially expressed genes between the ZH11 and KO based on RNA‐seq.

**Table S2** DGW1‐bound transcripts in PBR‐seq data.

**Table S3** DGW1‐bound transcripts in PBR‐seq data with high enrichment levels (Peak Fold Enrichment >10).

**Table S4** A list of primers used in this study.
