# Supplementary material for: DGW1, encoding an hnRNP‐like RNA binding protein, positively regulates grain size and weight by interacting with GW6 mRNA
Source: Plant Biotechnol J. 2023 Oct 20;22(2):512–26. doi: 10.1111/pbi.14202 (PMC10826988; doi:10.1111/pbi.14202)
Supplement: Supplementary file 1 — Figure S1 Other phenotypes of dgw1 mutant. Figure S2 Histocytological analysis of stem of WT and dgw1. Figure S3 Plant and panicle phenotypes of the DGW1‐CRISPR/Cas9 knockout plants. Figure S4 Histological analysis of the DGW1‐CRISPR knockout plant. Figure S5 Plant and panicle phenotypes of complemented transgenic lines. Figure S6 Bioinformatics analysis of DGW1 protein. Figure S7 mRNA and protein levels of DGW1 in OE lines. Figure S8 mRNA level and pre‐mRNA splicing efficiency of GW6 in WT and dgw1. Figure S9 Transient co‐expression of DGW1 and GW6 and polyribosomes‐bound GW6 mRNA analysis. Figure S10 Response of KO plants to BR and GA treatments. Figure S11 Heat map representing the transcript abundance of GA‐ and BR‐related differentially expressed genes in RNA‐seq. Figure S12 Multiple sequence alignment of AtUBP1b with its homologues in rice. Figure S13 Co‐localization of DGW1 with OsUBP1a/b. [file PBI-22-512-s001.pdf]

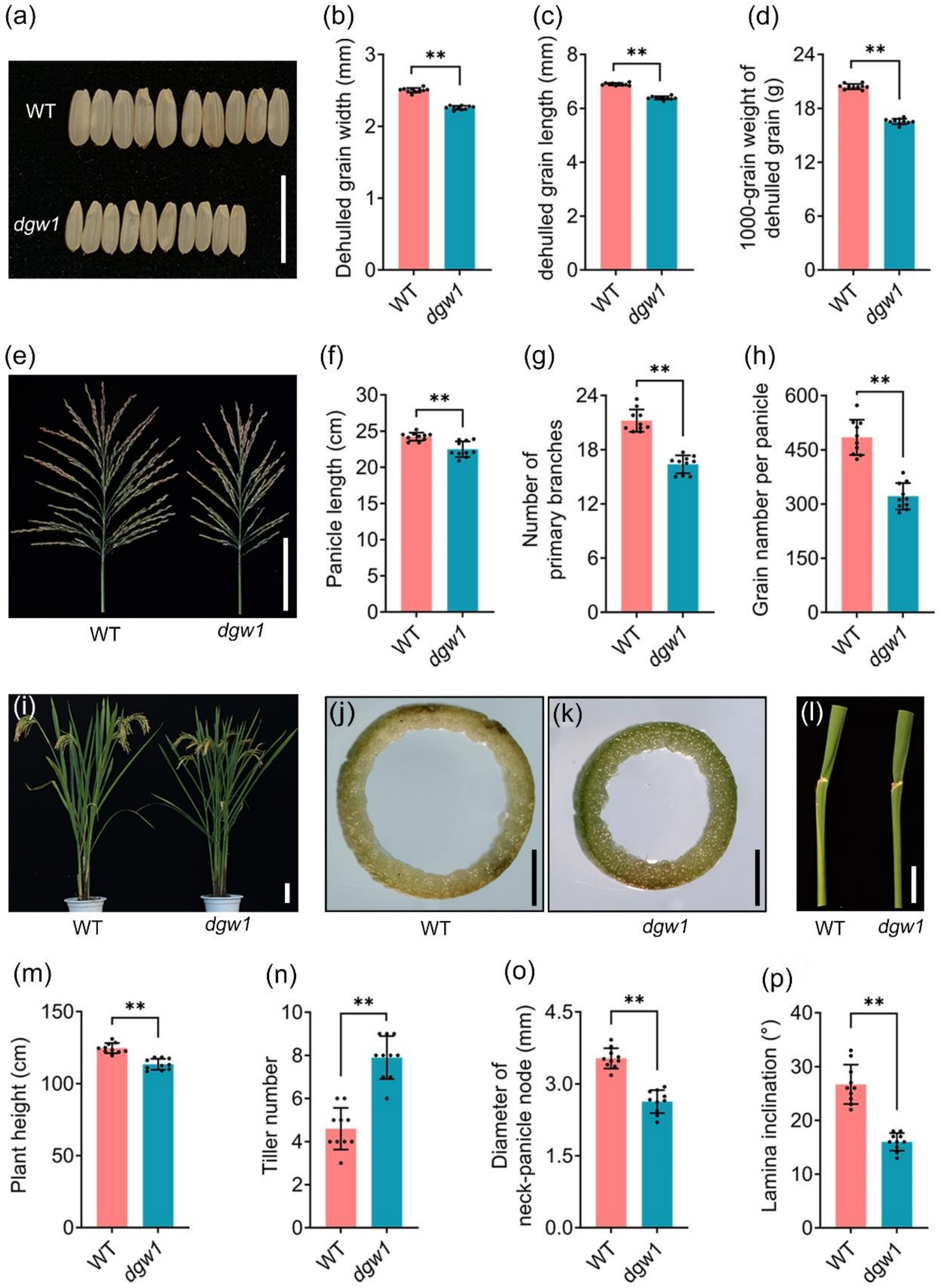

Figure S1. Other phenotypes of *dgw1* mutant.

(a) Dehulled grains of WT and *dgw1*. Bar = 1 cm

(b-d) Grain width (b), grain length (c) and 1000-grain weight (d) of WT and *dgw1* dehulled grain. Values are means ± SD (n = 10).

(e) Mature panicles from WT and *dgw1* rice plants. Bar = 10 cm.

(f-h) Comparisons between WT and *dgw1* for panicle length (f), number of primary branches (g), grain number per panicle (h). Values are means ± SD (n = 10).

(i) Plant architecture of WT and *dgw1* plants. Bar = 10 cm.

(j, k) Transverse sections of the neck-panicle node of WT (j) and *dgw1* (k). Bars = 1 mm.

(l) The lamina joint of flag leaf in WT and *dgw1*. Bar = 1 cm.

(m-o) Comparisons between WT and *dgw1* for plant height (m), tiller number (n), diameter of neck-panicle node (o). Values are means ± SD (n = 10).

(p) Quantification of lamina angle of the flag leaf. Values are means ± SD (n = 20)

** $P < 0.01$ compared with the wild-type using Student's t test.

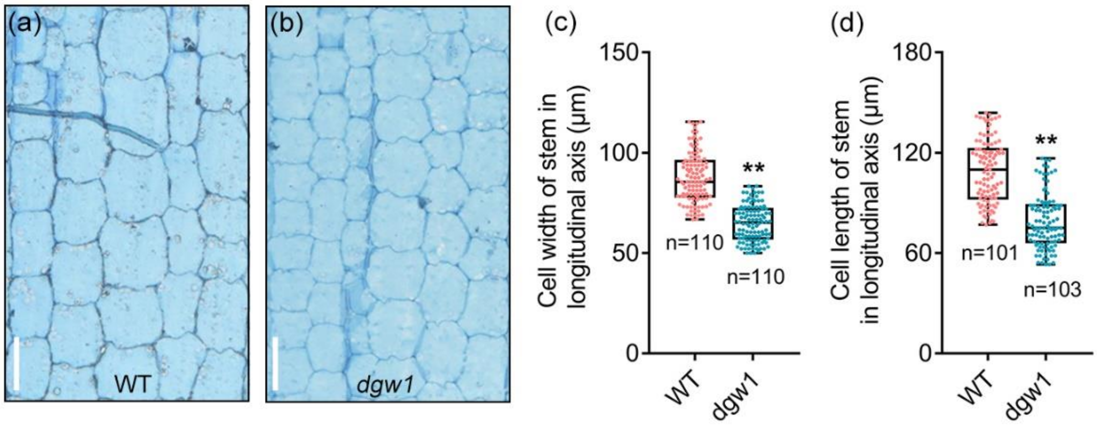

Figure S2. Histocytological analysis of stem of WT and *dgw1*.

(a, b) Longitudinal sections of the third internode of WT (a) and *dgw1* (b) stem. Bars = 100 μm.

(c, d) Comparison of cell width (c) and cell length (d) in the third internode between WT and *dgw1* mutant. Values are means ± SD ($n > 100$). ** $P < 0.01$ compared with the wild-type using Student's t test.

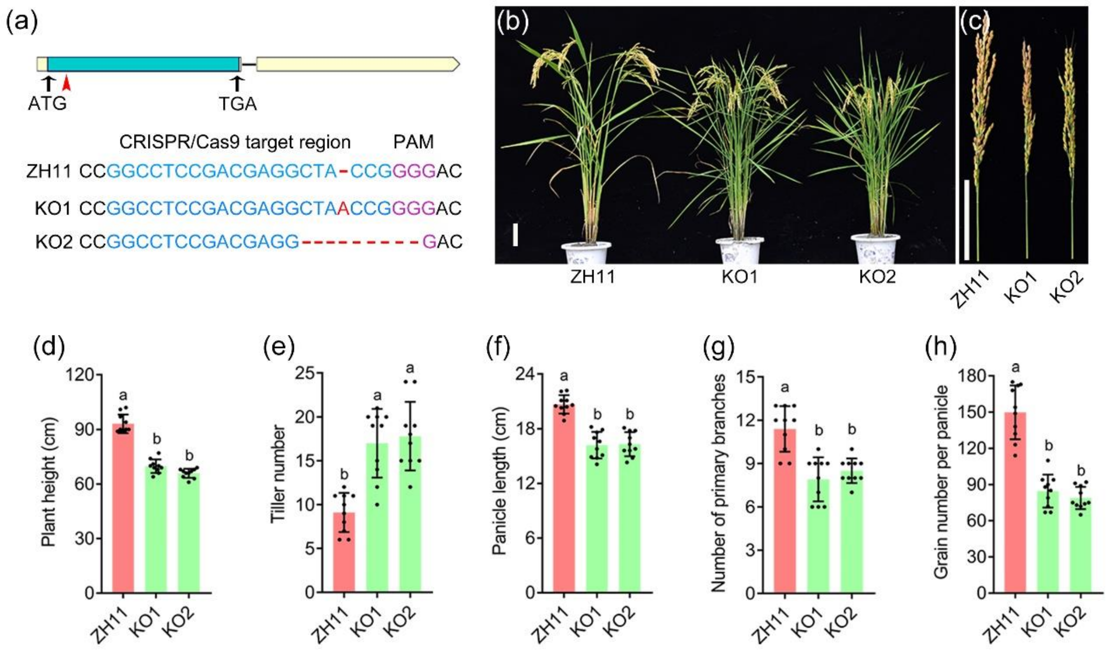

Figure S3. Plant and panicle phenotypes of the *DGW1*-CRISPR/Cas9 knockout plants.

(a) The diagram of CRISPR/Cas9 target site, and the mutated nucleotide sequences of KO1 and KO2 lines. The red arrow indicates the target site. The KO mutation is highlighted with red color.

(b, c) Plant (b) and panicle (c) phenotypes of ZH11 and two *DGW1* knockout plants. Bars = 10 cm.

(d-h) Comparison of plant height (d), tiller number (e), panicle length (f), number of primary branches (g), and grain number per panicle (h) between ZH11 and two *DGW1* knockout plants. Values are means ± SD (n = 10). Different lowercase letters indicate significant differences between ZH11 and KO lines (Student's t test, P < 0.05).

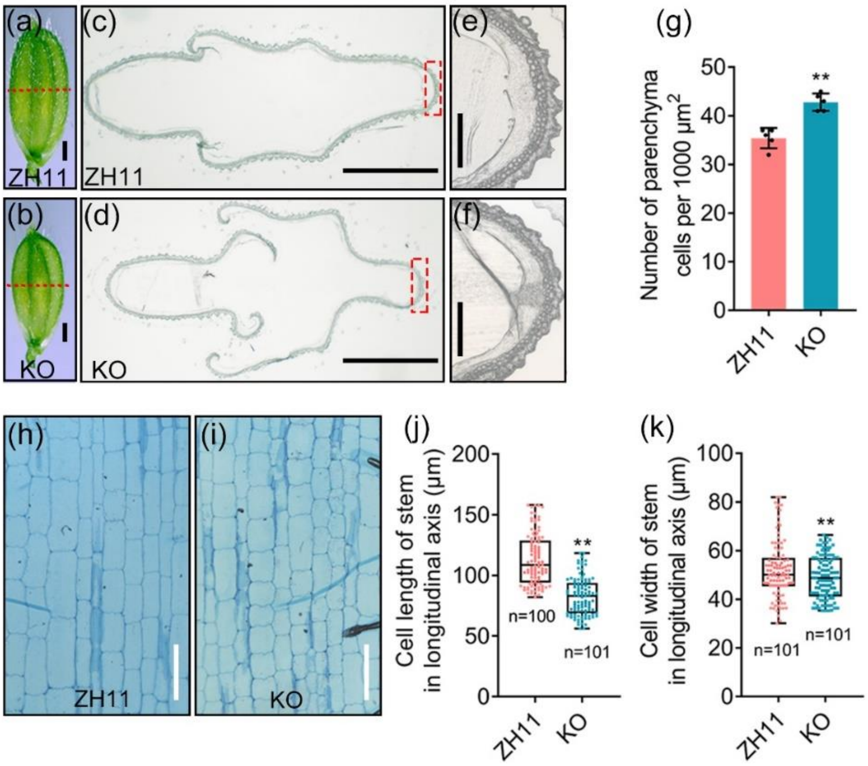

Figure S4. Histological analysis of the *DGW1*-CRISPR/Cas9 knockout plant.

(a, b) Spikelets of ZH11 (a) and KO (b) before anthesis. The red line indicates the cross-section position. Bars = 1 mm.

(c, d) Cross-sections of ZH11 (c) and KO (d) spikelet hulls. Bars = 1 mm.

(e, f) Magnified views of the cross-sections boxed in (c) and (d), respectively. Bars = 100 μm.

(g) Number of parenchyma cells per 1000 μm^2 in ZH11 and KO. Values are means ± SD ($n = 5$).

(h, i) Longitudinal sections of the third internode of ZH11 (h) and KO (i) stem. Bars = 100 μm.

(j, k) Comparison of cell length (j) and cell width (k) of longitudinal sections in the third internode between ZH11 and KO. Values are means ± SD ($n \geq 100$).

** $P < 0.01$ compared with the ZH11 using Student's *t* test.

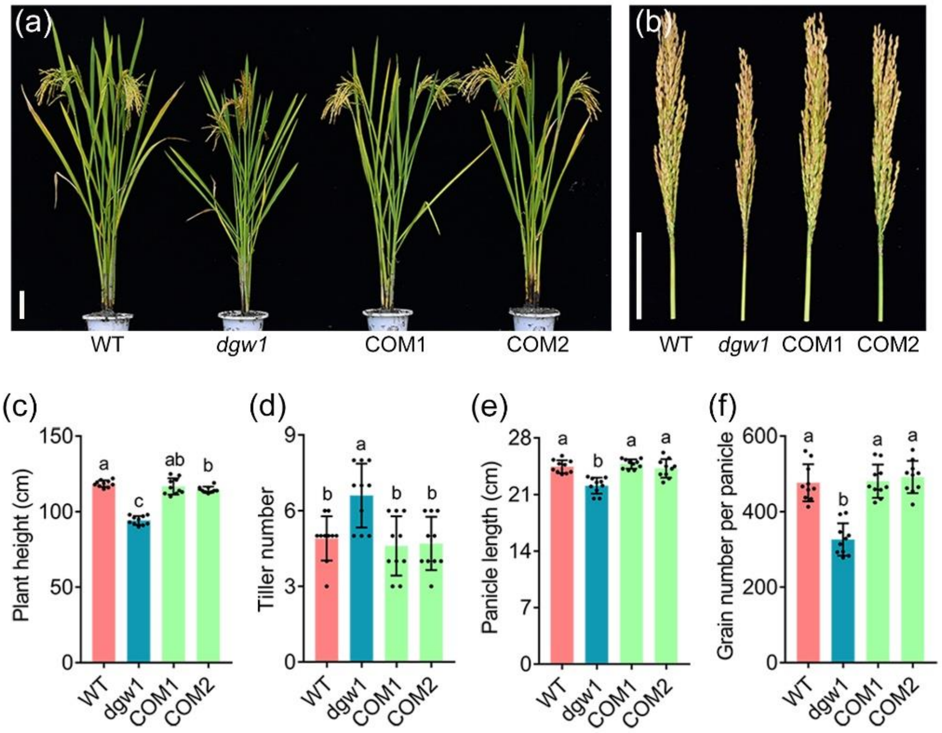

Figure S5. Plant and panicle phenotypes of complemented transgenic lines.

(a, b) Plant (a) and panicle (b) phenotypes of WT, *dgw1* and two complemented transgenic lines. Bars = 10 cm.

(c-f) Comparison of plant height (c), tiller number (d), panicle length (e), and grain number per panicle (f) in WT, *dgw1* and two complemented transgenic lines. Values are means ± SD (n = 10). Different lowercase letters indicate significant differences between WT and other lines (Student's t test, $P < 0.05$).

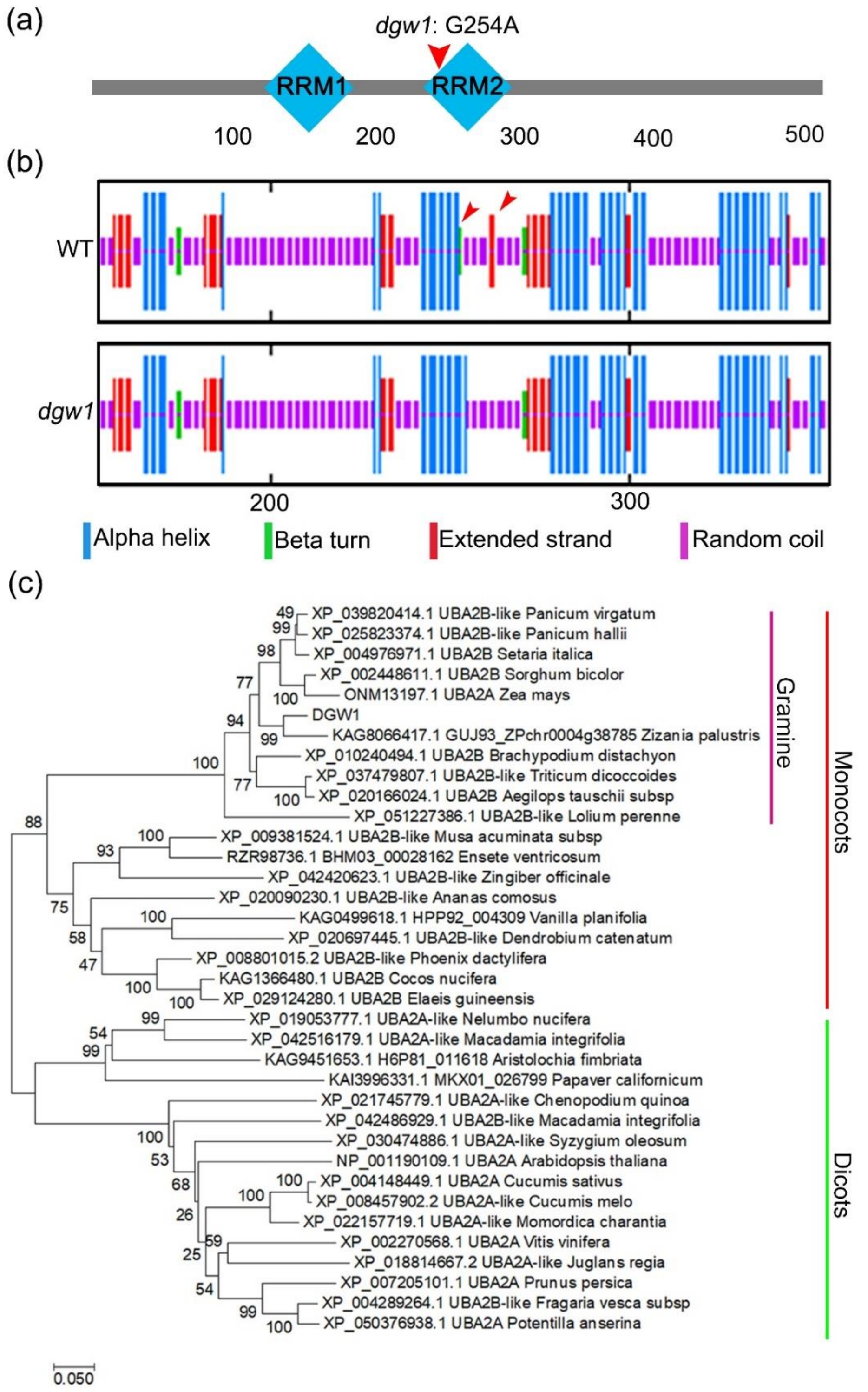

Figure S6. Bioinformatics analysis of DGW1 protein.

(a) Schematic diagram of DGW1 protein structure. The red arrow indicates the mutation site.

(b) Predicted model of DGW1 protein in the WT and *dgw1*. The red arrows show where there are mutations in the protein structure. The two-dimensional protein

structures were predicted using SOPMA (http://npsa-pbil.ibcp.fr/cgi-bin/npsa_automat.pl?page=npsa_sopma.html).

(c) Phylogenetic tree of DGW1 and its orthologues in plant species. Multiple sequence alignment of these proteins was performed using the ClustalW program in the MEGA7 software, and the phylogenetic tree was constructed using the neighbor-joining method with 1,000 bootstrap replicates. Scale bar indicates number of amino acid changes per site. Numbers on branches are the bootstrap support values of each clade.

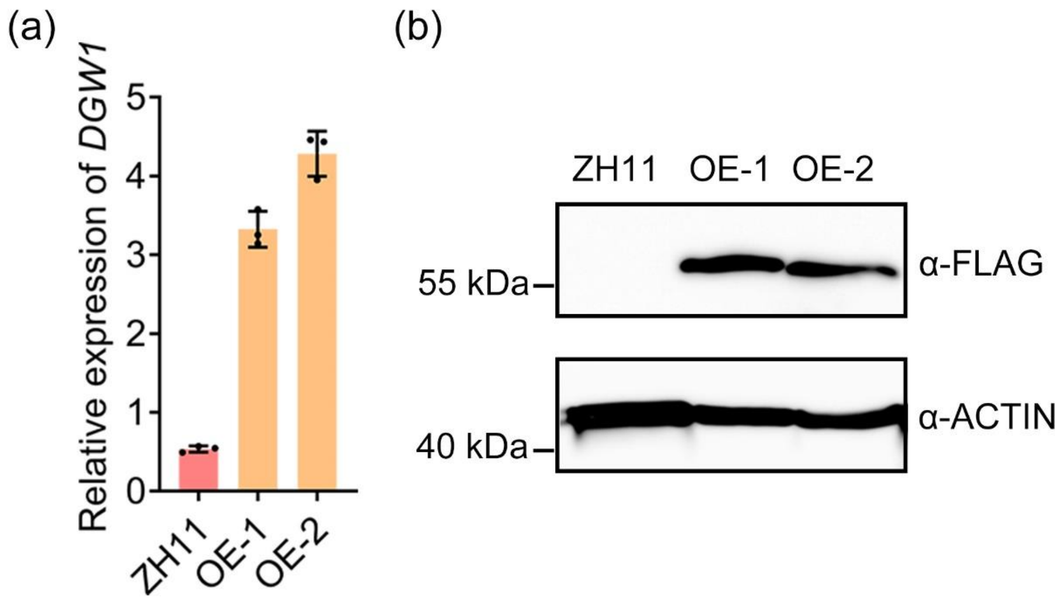

Figure S7. mRNA and protein levels of *DGW1* in OE lines.

(a) The expression levels of *DGW1* in ZH11 and OE lines were analyzed by RT-qPCR. Rice *ACTIN* gene was used as a control. Values are means ± SD (n = 3).

(b) Western blotting analysis showed DGW1-FLAG protein levels in ZH11 and OE lines.

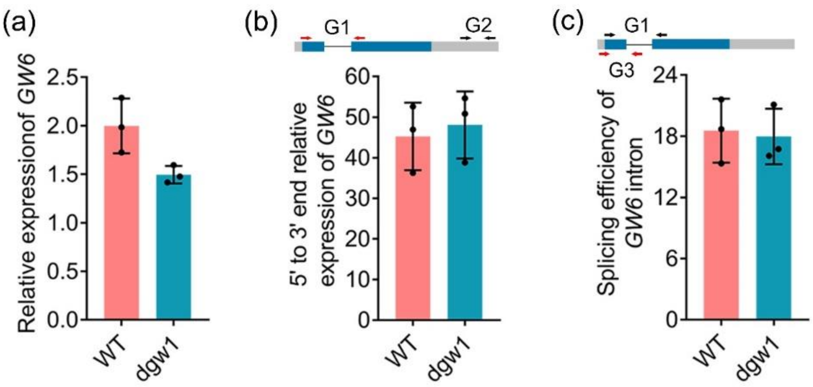

Figure S8. mRNA level and pre-mRNA splicing efficiency of *GW6* in WT and *dgw1*.

(a) RT-qPCR analysis of *GW6* mRNA level in panicles of WT and *dgw1*. Values are means ± SD (n = 3).

(b) *GW6* mRNA turnover rates in WT and *dgw1*. The mRNA turnover rates were calculated as the ratio of the 5′- end to the 3′- end. Primer (G1 and G2) position is indicated with arrows on the *GW6* gene structure in the top panel. Values are means ± SD (n = 3).

(c) Splicing efficiencies of the *GW6* gene in the panicles of WT and *dgw1* by RT-qPCR validation. Black arrows indicate the primers (G1) for detecting the spliced events. Red arrows represent the primers (G3) for detecting the unspliced events. The splicing efficiency was calculated as the ratio of spliced *GW6* RNA to the level of unspliced *GW6* RNA.

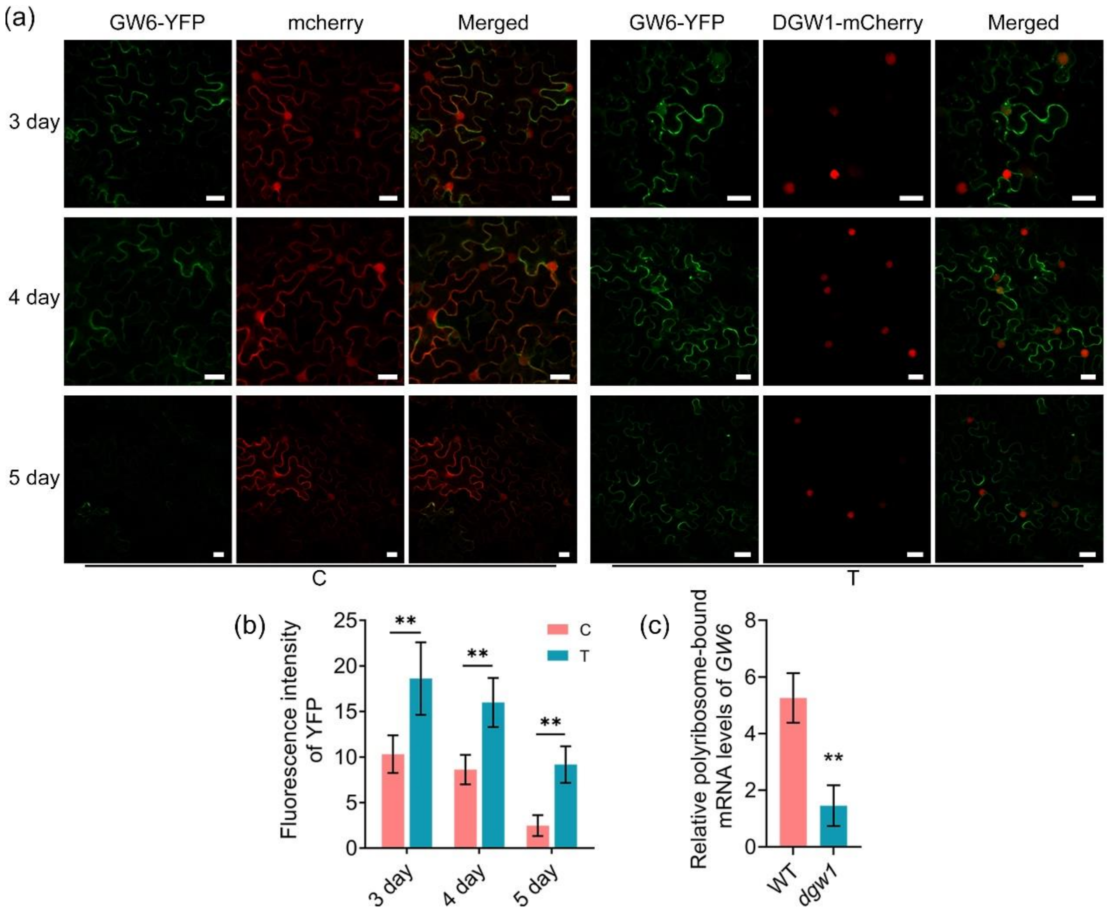

Figure S9. Transient co-expression of *DGW1* and *GW6* and polyribosomes-bound *GW6* mRNA analysis.

(a) Fluorescence intensity and degradation time course of GW6-GFP fusion proteins when co-expressed with DGW1-mCherry or mCherry in *N. benthamiana* leaves. C, control group; T, treatment group. Bars = 50 μm.

(b) Summary data for averaged mean fluorescence intensity from GW6-YFP (right). ** $P < 0.01$, Student's t test ($n = 10$ cells).

(c) RT-qPCR assays show the abundance of *GW6* mRNA in polyribosome from WT and *dgw1* plants. Values are means ± SD ($n = 3$). ** $P < 0.01$, Student's t test.

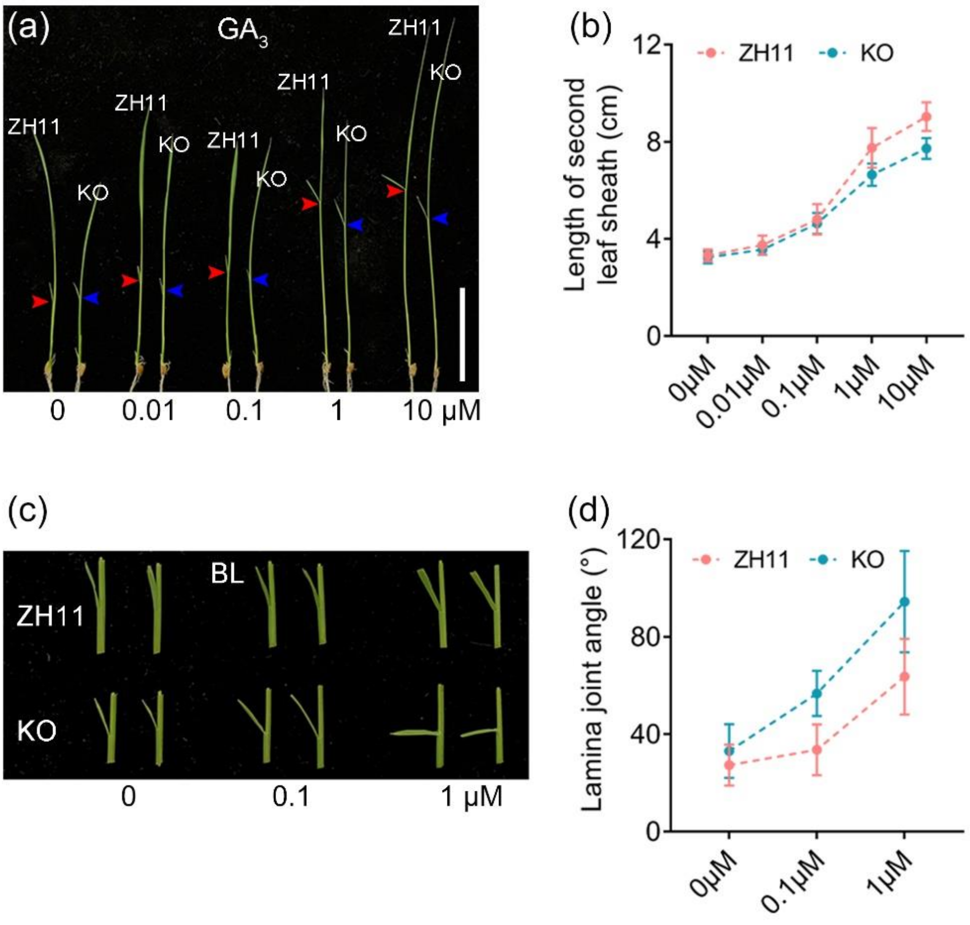

Figure S10. Response of KO plants to BR and GA treatments.

(a) Dose-dependent responses of ZH11 and KO seedlings to GA_3 treatment. The red and blue arrows indicate the second leaf of ZH11 and KO, respectively. Bar = 5 cm.

(b) Comparison of second leaf sheath length between ZH11 and KO with GA_3 treatment. Data are means ± SD (*n* = 20).

(c) The leaf inclination of ZH11 and KO in the presence of indicated concentration of 24-epiBL.

(d) Statistical analysis of leaf inclination in (c). Values are means ± SD (*n* = 20).

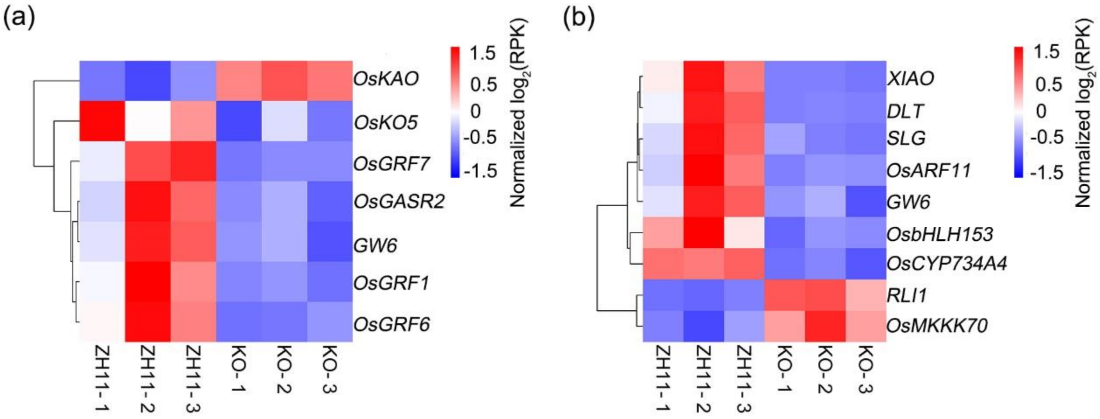

Figure S11. Heat map representing the transcript abundance of GA- and BR-related differentially expressed genes in RNA-seq.

(a) Transcript abundance of *OsKAO*, *OsKO5*, *OsGRF7*, *OsGASR2*, *GW6*, *OsGRF1* and *OsGRF6* in ZH11 and KO seedlings.

(b) Transcript abundance of *XIAO*, *DLT*, *SLG*, *OsARF11*, *GW6*, *OsbHLH153*, *OsCYP734A4, RLI1* and *OsMKKK70* in ZH11 and KO seedlings.

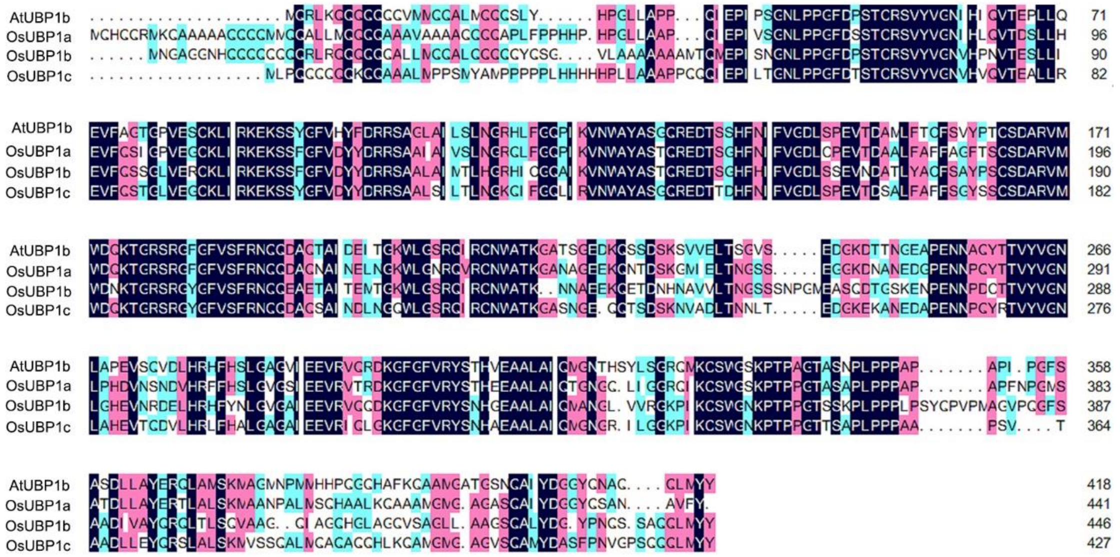

Figure S12. Multiple sequence alignment of AtUBP1b with its homologues in rice. Identical amino acids are shaded in black; 75% or 50% similar amino acids are shaded in red or blue, respectively.

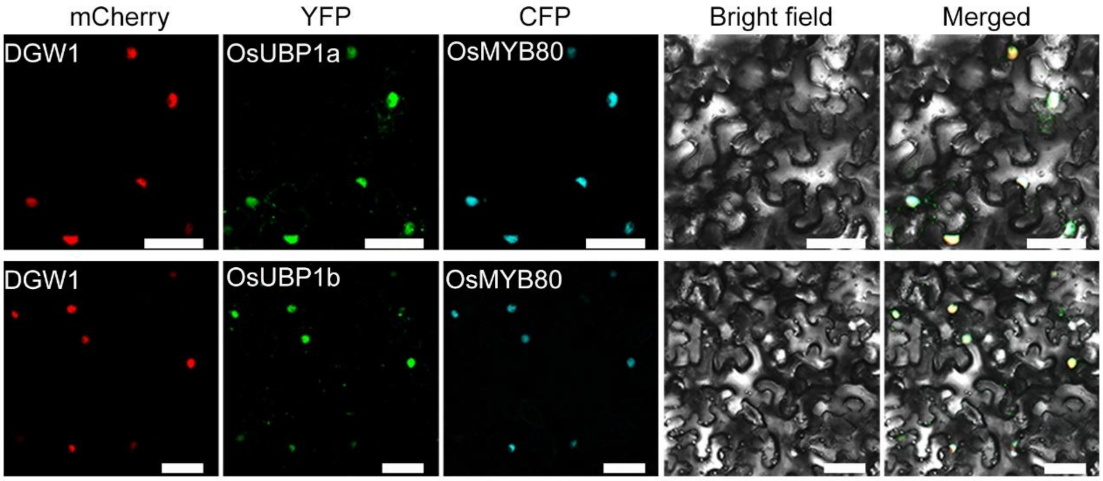

Figure S13. Co-localization of DGW1 with OsUBP1a/b.

Laser confocal microscope analysis showed that DGW1 co-localized with OsUBP1a/b in the nucleus of *N. benthamiana* leaf cells. OsMYB80-CFP was used as a nuclear marker. Bars = 50 μm.
